# From Functional Nanomaterials to Trustworthy Wearable Sensing Systems: A Cross-Layer Review of Bioelectronic Interfaces, Wireless and Power Integration, Artificial Intelligence Inference/Interpretation, and Human-Centred Validation

**DOI:** 10.3390/s26216877

**Published:** 2026-10-30

**Authors:** Hyejeong Choi, Neha Suvindran, Morteza Lotfi Neyestanak, Yi-Chieh Wang, Menglei Hu, Gongbo Yang, Peyman Servati

**Affiliations:** 1 Flexible Electronics and Energy Lab (FEEL), Department of Electrical and Computer Engineering, The University of British Columbia, 5500-2332 Main Mall, Vancouver, BC V6T 1Z4, Canada; hchoi2@ece.ubc.ca (H.C.); nehas@ece.ubc.ca (N.S.); lotfiney@ece.ubc.ca (M.L.N.); yichiehwang@alumni.ubc.ca (Y.-C.W.); gongboyang@ece.ubc.ca (G.Y.); 2 Texavie Technologies Inc., 148-970 Burrard St, Vancouver, BC V6Z 2R4, Canada; 3 Flexible Electronics and Energy Lab (FEEL), School of Biomedical Engineering, The University of British Columbia, 6088 University Boulevard, Vancouver, BC V6T 1Z3, Canada

**Keywords:** functional nanomaterials, flexible and wearable sensors, bioelectronic interfaces, wireless sensing, multimodal sensing, machine learning, physiological monitoring, human–machine interaction, human-centred validation, claim–evidence alignment

## Abstract

Material-level sensitivity or conductivity alone does not determine whether a wearable sensor produces trustworthy data. The signal must survive body coupling, packaging, wireless transfer, power constraints, and algorithmic interpretation. This cross-layer critical review examines six connected evidence streams: functional nanomaterial platforms, epidermal and implantable bioelectronic interfaces, wireless readout and power, integrated wearable platforms, artificial intelligence (AI)-based interpretation, and human-centred applications. Hysteresis and drift, interface instability, motion and environmental exposure, readout and power limits, placement and calibration dependence, and inter-user variability can degrade information across these layers. Robustness testing and claim-matched validation are needed to establish what the resulting outputs support. We therefore compare technologies through the failure modes and evidence requirements that persist after integration, rather than through isolated peak metrics. The synthesis offers conditional material-selection criteria for specific device roles, distinguishes demonstrated component functions from application-level claims, and identifies the reporting conditions and use-dependent validation priorities needed to interpret those claims.

## 1. Introduction

### 1.1. Motivation and Unmet Need

Wearable sensing has progressed beyond the question of whether electronics can conform to the body. The harder problem is whether a device can preserve useful information during actual use. Epidermal electronics established that ultrathin devices can follow skin mechanics [1], while integrated sweat-monitoring systems combined sensing with calibration, signal conditioning, and wireless communication [2]. These advances highlight a central distinction: sensitivity measured at a transducer under defined conditions does not by itself establish the validity of the resulting measurement. Body coupling, environmental exposure, and calibration can change the relationship between the sensed input and reported output, making validation against an appropriate reference under intended-use conditions essential.

These requirements vary across applications. Skin-interfaced electrophysiological monitoring requires stable contact and motion-artifact control [3], whereas biochemical sensing depends on selectivity and calibration as biofluid conditions vary [2]. For motion and tactile systems, sensor placement, deformation history, and differences between users influence how measurements should be interpreted. Where sensing informs assistive feedback, the timing and usability of that feedback introduce additional validation requirements. These dependencies motivate a cross-layer critical review of how material and device capabilities translate into integrated measurements and what evidence supports the resulting application claims.

### 1.2. Why Material–Device–System Co-Design Is Needed

Layer-specific advances can be substantial, but their system-level significance depends on how components interact under operating conditions and how the resulting outputs are validated for their intended use. Ma et al. reported a high peak gauge factor in a layered MXene (a two-dimensional transition-metal carbide) compression sensor, alongside declining sensitivity and a response approaching saturation at higher strain [4]. In an integrated sweat platform, temperature compensation corrected glucose and lactate overestimation as solution temperature increased [2]. Together, these findings highlight why the operating range and calibration must be considered when interpreting the reported measurement.

These dependencies extend through the body interface, wireless link, and power supply. Carbon-nanotube (CNT)-fibre electrodes with 4 µm parylene-C insulation supported single-unit recording in freely moving rats for up to 3–4 months, compared with approximately two weeks using 2 µm; accompanying impedance changes were consistent with leakage [5]. A deformation-responsive dielectric reduced strain-induced resonance shifts during skin-interfaced wireless operation [6], illustrating how body mechanics influence communication design. Approximately 60 h of in vitro charge–discharge with replenished fuel, alongside separate on-body sensing [7], further highlights the need to evaluate available energy and complete-device workload when assessing sustained autonomy.

Beyond integration, the strength of an application claim depends on the reference, population, and intended outcome. Concurrent Vicon motion-capture measurements supported indoor gait agreement, while outdoor measurements were compared with references acquired at a different time [8]; concurrent outdoor testing would strengthen claims of accuracy in that setting. With the same model and preprocessing, activity-recognition accuracy was 99.85% under random 10-fold cross-validation and 85.1% under leave-one-subject-out evaluation [9], demonstrating the importance of unseen-user testing without attributing the difference to data leakage. Stiffness-recognition and vibrotactile-feedback studies involving participants with and without amputation [10] extended evaluation to relevant users and tasks, while broader functional benefit warrants corresponding outcome measures.

We therefore treat trustworthy sensing as a cross-layer property, not as an isolated material, device, or algorithm metric. For each layer, we ask four questions: what function is established, what degrades the information after integration, which design or analytical choices suppress that degradation, and what evidence is required before a stronger system- or application-level claim is justified.

### 1.3. Positioning Relative to Existing Reviews

**Figure 1:**
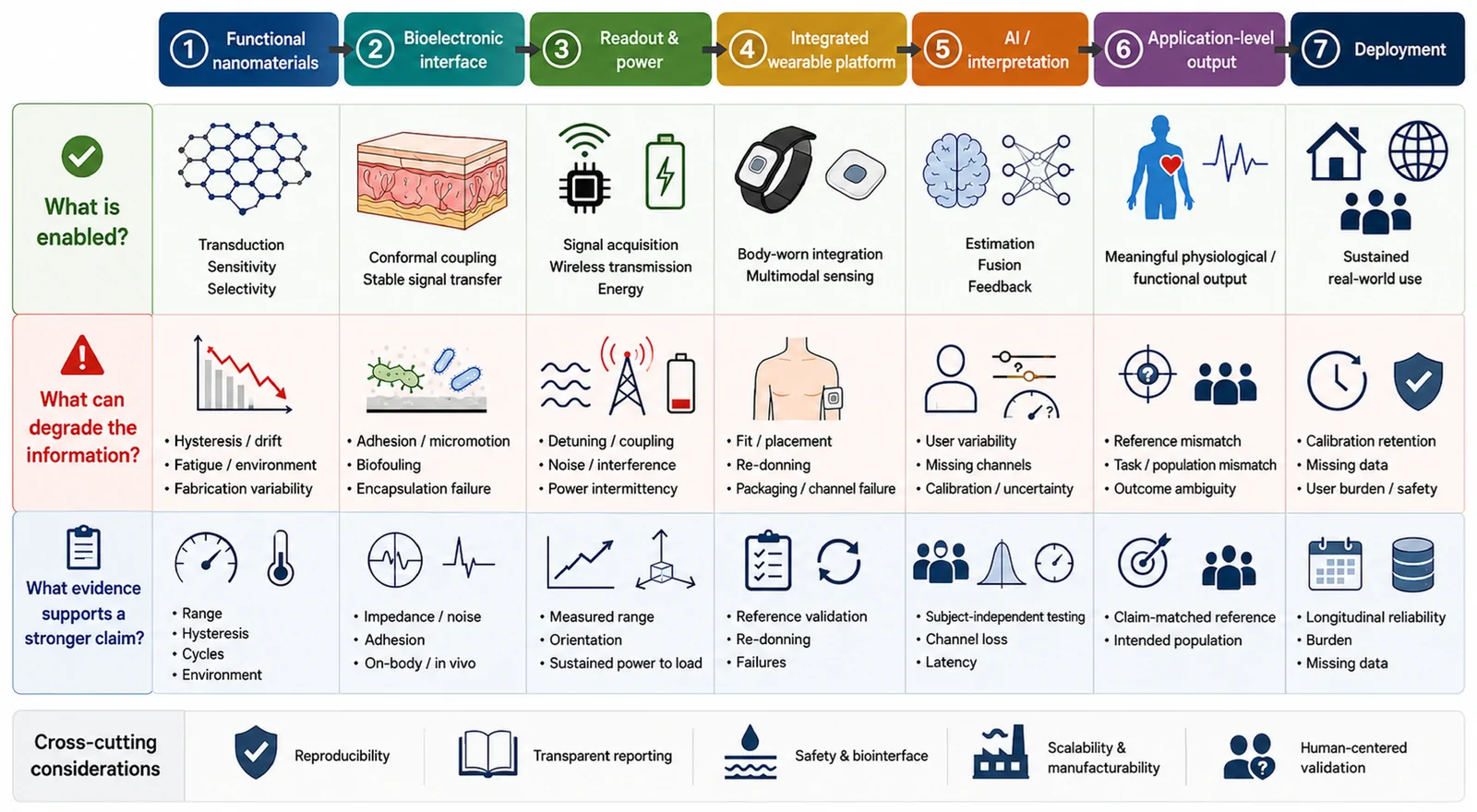
Cross-layer framework for functional nanomaterial-enabled wearable sensing. The sensing pathway progresses from functional nanomaterials through the bioelectronic interface, readout and power, integrated wearable platform, artificial intelligence (AI)-enabled interpretation, application-level output, and deployment. For each layer, the figure summarizes the function enabled, major sources of information degradation, and evidence needed to support stronger downstream claims. Cross-cutting considerations include reproducibility, transparent reporting, safety and biointerface requirements, scalability and manufacturability, and human-centred validation.

Recent reviews have explored functional materials, biointerfaces, wireless integration, machine-learning interpretation, and clinical translation, often spanning several of these areas [11,12,13,14,15,16,17,18]. Building on these contributions, we follow the sensing pathway to examine how operating conditions and constraints at one layer influence downstream measurements and their interpretation (Figure 1). We then consider what the available evidence supports at the integrated-system and intended-use levels.

Table 1 positions this analytical emphasis among eight selected reviews. It summarizes their stated scopes and areas of coverage to clarify differences in emphasis, without ranking review quality or implying that topics not highlighted here were overlooked.

By making these distinctions explicit, this review links use-dependent material selection and service windows (Table 2) to the interpretation of application-level claims (Section 8), reporting requirements (Section 9), and priorities for further validation (Section 10).

### 1.4. Scope and Contributions of This Review

The review spans the material-to-application pathway, from conductive, electrochemical, optical, ionic, and textile-compatible nanomaterials through epidermal and implantable biointerfaces, wireless and passive readout, power integration, and machine-learning interpretation. It considers physiological and biochemical monitoring, movement and tactile sensing, assistive feedback, and rehabilitation, including sensor fusion and personalization. Implantable and preclinical studies are included where they inform transferable questions about interfacing, packaging, telemetry, durability, or validation.

Functional nanomaterial-enabled denotes systems in which nanoscale or nanostructured materials contribute directly to sensing, biointerfacing, electrical or optical transduction, readout, energy conversion, or another signal-path function. Human-centred sensing refers to evaluating performance in relation to body coupling, user variability, functional relevance, calibration and interaction burden, and intended-use conditions. These definitions distinguish material function from evidence supporting application-level claims.

Epidermal electrophysiology depends on skin–electrode contact under motion [3], with sweat exposure and repeated attachment warranting assessment when relevant. Implantable interfaces pose different function- and duration-dependent questions. Rat neural recordings illustrate insulation-related stability [5], while tissue response and micromotion call for separate evaluation.

Textile sensing depends on fit and fabric deformation [19]. Knittable, washable yarns demonstrate material-level processing feasibility [20], while garment reliability during wear and after washing, manufacturing reproducibility and production cost require separate evidence. For accessories, placement and readout must be linked to the interpreted task output. Wireless-mask monitoring illustrates readout [21], whereas joint-kinematic estimation from inertial data provides transferable interpretation evidence [22]. Appropriate reference tests should reflect intended placement, calibration and, where applicable, wireless conditions.

## 2. Review Approach and Evidence Synthesis

### 2.1. Review Scope and Analytical Framework

This article is a structured, evidence-informed critical review with a translational roadmap. It does not attempt to estimate a pooled intervention effect or exhaustively enumerate every paper in the field. Across six evidence streams, we examine reported functions and limitations from material characterization to application validation, assessing downstream claims in light of operating conditions and the available validation evidence.

Section 3, Section 4, Section 5, Section 6, Section 7 and Section 8 were developed as topic-specific evidence streams, with search strategies adapted to each domain’s terminology and indexing practices. The synthesis examined the functions demonstrated and limits reported, considered whether mitigations were proposed or tested, and evaluated the evidence available for integrated-system and intended-use claims.

Artificial intelligence (AI)-assisted tools supported literature organization, preliminary relevance assessment, consistency checks, preparation of selected figures using published results, and manuscript editing. Where full-text verification was documented, the authors checked relevant quantitative comparisons against the cited sources. Final decisions on source selection, citations, scientific interpretation, and conclusions remained with the authors.

### 2.2. Literature Identification and Source Selection

**Figure 2:**
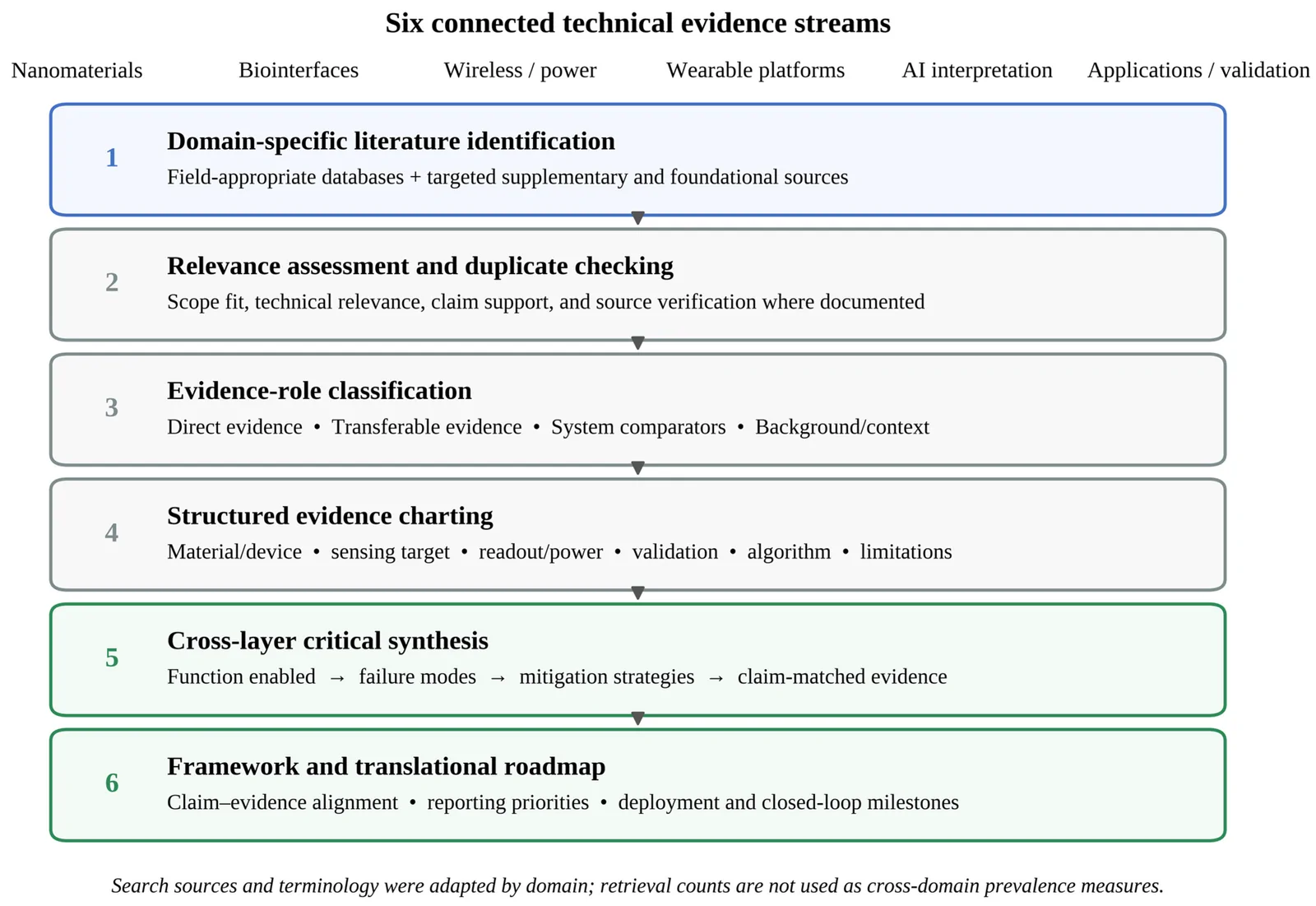
Structured literature-identification and cross-layer synthesis workflow used in this review. Domain-specific database searches and targeted supplementary searches feed relevance assessment, evidence-role classification, and data charting. The synthesis then maps established functions, failure modes, mitigation strategies, and validation evidence across the material-to-application pathway to derive the claim–evidence framework and translational roadmap.

The six technical streams were built primarily from English-language records within the documented coverage windows (Figure 2). Depending on the field, sources included Scopus, Web of Science Core Collection, IEEE Xplore, PubMed, Google Scholar, Semantic Scholar, OpenAlex, arXiv, and targeted supplementary searches. Supplementary Table S1 lists the information sources, search dates, coverage windows, and limits. Supplementary Search Documentation S1A–S1F preserves the available section-specific search strings and search logic. We did not reconstruct search details that were absent from the original contributor records.

The six streams used domain-specific literature pools because indexing coverage, publication formats and terminology differ across materials, bioelectronics, radio frequency (RF) systems, wearable platforms and AI. Duplicate checking and relevance triage were applied where the source records allowed them. Foundational papers, targeted contextual studies and technology-disclosure sources were added when they resolved a specific cross-layer or translational question. Neither the pools nor these additions were used to estimate field prevalence.

### 2.3. Evidence Selection and Evidence Roles

A source was retained when it informed at least one layer of the review: nanomaterial-enabled sensing; flexible, stretchable, textile-integrated, epidermal, implantable, or otherwise soft devices; bioelectronic interfaces; wireless or passive readout; power delivery or harvesting; machine-learning interpretation; or human-centred sensing applications. Supplementary Table S1 records the publication-type limits applied to the documented searches; these do not constitute a uniform restriction on the types of sources cited in the final cross-stream synthesis. Section 7, for example, includes peer-reviewed conference papers because machine-learning and robotics results are often reported first in that format.

The evidence spans material and component characterization, integrated-device tests, bench and proxy studies, acute and chronic in vivo work, on-body human validation, intended-user or patient studies, and real-world or longitudinal deployment. We excluded sources that did not address the sensing, device, interpretation, or human-centred question relevant to the stream, duplicated another source, or lacked enough technical detail to support the claim under discussion. Non-peer-reviewed material appears only as explicitly labelled background or technology context; it is not treated as equivalent to peer-reviewed primary evidence. Patent publications are used only to document architecture, integration, manufacturing, or implementation pathways.

Detailed discussion was prioritized by cross-layer relevance, technical content, validation detail, representativeness of a major architecture or failure mode, and direct support for the claim being made. We classify evidence into four roles: direct evidence contains a nanomaterial or nanostructured component in the wearable or bioelectronic signal path; transferable evidence comes from adjacent systems that resolve an interface, calibration, robustness, or integration problem; system comparators establish wearable validation or deployment requirements without being primarily nanomaterial-enabled; background sources provide reviews, foundational results, or methodological context. These categories describe how a source is used; they do not imply equal translational strength.

### 2.4. Data Charting and Critical Thematic Synthesis

For each source selected for detailed synthesis, we charted bibliographic provenance, material and device characteristics, sensing mechanism and target, body placement or use setting, performance metrics, readout and power architecture, validation population and reference, algorithm type where applicable, key findings, and limitations. Stream-specific fields were added only when the technical question required them. Bioelectronic studies, for example, were charted for interface architecture and chronicity; wearable platforms for deployment context and intended-user status; and AI studies for evaluation split, calibration burden, channel or modality loss, latency, compute location, uncertainty handling, and feedback evidence.

Quantitative, participant-level, and clinical statements used in the text, figures, or tables were checked against the cited source whenever full-text verification was documented by the section team. Unresolved details were not filled by inference. Because sensing modality, outcome definition, reference method, population and deployment setting differ across studies, we did not pool quantitative results. Instead, we used the charted conditions and limitations to assess claims along the material-to-application pathway and derive reporting needs and priorities for further validation.

## 3. Functional Nanomaterial Platforms

Material selection depends on the target signal, anticipated deformation or exposure, and required duration. A service window describes the demonstrated conditions over which a specific formulation and device retain a defined function, rather than a lifetime attributable to the entire material class.

Sensing signals arise through overlapping chemical, structural and physical mechanisms. Surface and terminal groups, redox activity and molecular recognition contribute to analyte-dependent responses, while conductive contacts, nanoscale morphology, porosity and fibre or yarn geometry shape mechanical transduction. Electronic and ionic transport, fluorescence, photoactivity and fluidic metallic behaviour provide additional pathways. Hybrid architectures combine these contributions, making formulation and processing part of the transduction design.

The studies that follow cover representative material families, but the relevant comparison is between their intended device functions and the evidence supporting those functions. A material can serve as a deformation-sensitive element, a conductor intended to remain stable, or part of an optical or ionic interface. Its response must therefore be interpreted in relation to the actual test conditions and fabrication route. These distinctions underpin the application-specific material-selection and verification priorities in Table 2.

### 3.1. Carbon-Based Nanomaterials and Conductive Composites

Carbon-based nanomaterials—including graphene, graphene oxide, reduced graphene oxide (rGO), carbon nanotubes, carbon black, carbonized fibres, and hybrid carbon composites—are used in flexible pressure, strain, tactile, and textile sensors. Their conductivity and compatibility with elastomers, hydrogels, paper, fabric, and printable inks depend on formulation and processing; low density and chemical tunability also motivate their use [23,24,25,26]. In piezoresistive designs, applied strain or pressure alters tunnelling distances, interparticle contacts, crack geometry, or percolation pathways, thereby modulating resistance [19,27].

The network that carries current must also respond reproducibly to deformation. Graphene and CNTs can establish conductive pathways at relatively low filler loading, while CNTs can bridge local cracks during stretching. Conductive-network architecture, filler dispersion, and reduction of graphene oxide influence electrical response [24]. Carbon black offers a lower-cost alternative to graphene [24], while soaking enables carbon-black-coated sponge pressure sensors [28]. After textile integration, repeated deformation and washing become additional conditions for retaining the calibrated response [19].

Lv et al. provide a within-study comparison of graphene-assisted and hydrogel-only strain sensing. At 25% tensile strain, the graphene/water–glycerol hydrogel reached a gauge factor of approximately 2.4, compared with 0.59 for the bare hydrogel [27]. The stretch–release response was nearly symmetric, the graphene layer remained attached after ten stretches at that strain, and glove-mounted sensors responded to finger-joint bending [27]. These tests support initial wearable sensing, while quantitative joint-angle tracking would require reference-based calibration across the intended movement range and evaluation after repeated attachment and exposure.

### 3.2. MXenes and Other Two-Dimensional Nanomaterials

MXenes provide conducting layered structures with tunable surface chemistry and morphology suitable for different sensing mechanisms [29,30]. Ti_3_C_2_T_x_ is the dominant MXene example in the literature considered here. Its metallic conductivity, hydrophilicity, and -O, -OH, and -F surface terminations permit solution processing into films, fibres, aerogels, coatings, and composites [29,30,31,32]. Reported devices span pressure, strain, humidity, sweat chemistry, and multimodal sensing [30,31,33,34,35].

Mechanical MXene sensors exploit changes in interlayer spacing, contact resistance or conductive-network geometry, but the resulting response depends on how the device is loaded. In a multilayer Ti_3_C_2_T_x_ compression sensor, Ma et al. reported a peak gauge factor of 180.1 within the 0.19–0.82% compressive-strain range; sensitivity declined above 0.82% and the response approached saturation beyond 1.80% [4]. For tensile sensing, Cai et al. combined MXene nanostacks with crossing CNTs to obtain tunable sensing ranges spanning 30–130% strain [34]. These architectures illustrate why deformation mode and calibrated range must be considered alongside peak sensitivity.

The useful service window also depends on material preparation and the assembled device. In Ma et al., partially exfoliated MXene was less sensitive than well-layered material, while the finished sensor combined a polyimide electrode substrate, a protective fabric layer and polydimethylsiloxane (PDMS) encapsulation [4]. Its signal showed little attenuation after 4000 compression cycles at 7.5 kPa [4]. In textile routes, industrial knitting and separate washing tests of MXene-coated cotton yarns [20] and 3D printing of MXene–cellulose nanofibril inks [36] address different processing demands. Sweat-pH sensors based on functionalized MXene–polyaniline composites use superhydrophobic protection to improve stability in wet environments [31,35]. Together with formulation-dependent oxidation concerns [29,30], these results direct evaluation toward the relevant mechanical, washing or electrochemical exposure rather than assuming transferable MXene durability.

### 3.3. Perovskites, Quantum Dots, and Optical Nanomaterials

Optical nanomaterials extend sensing beyond changes in electrical resistance, but their functional roles differ between photodetection and fluorescence-based chemical detection. Perovskites and quantum dots (QDs) offer tunable absorption and emission, supporting flexible photodetectors and luminescent sensors [37,38,39,40,41]. Zheng et al. combined a reticulated single-walled carbon-nanotube (SWNT) network with CsPbI_3_ QDs: the nanotubes supplied mechanical extensibility, while the QDs enhanced light absorption and photoresponse relative to SWNT-only devices. The hybrid device also underwent repeated bending and stretching tests [40].

Optical materials must also be integrated with the intended geometry and measurement medium. Wu et al. used electrospinning at 40 ± 5 °C to form photoresponsive CsPbI_2_Br/polymer nanofibers on yarns, demonstrating detection of intense pulsed light [42]. Li et al. immobilized luminescent QDs on a polymer tip at the end of an optical fibre and measured ZnO-nanoparticle-dependent quenching in ethanol [43]. These studies establish different forms of fibre-integrated optical sensing. For a physiological application, however, the relevant optical path (including tissue effects where applicable), source–detector coupling and measurement reference would require application-specific evaluation.

Mechanical and storage tests further define what the optical evidence supports. In CsBr/KBr-treated CsPbBr_3_-QD photodetector arrays, Shen et al. reported approximately 94% retention of photo-induced current at a 60° bend and approximately 93% retention after 1600 bending cycles in self-powered operation [44]. The authors separately reported little change in photoresponse after more than seven months of ambient storage [44]. These tests demonstrate detector stability under specified conditions, but do not establish physiological measurement accuracy or prolonged wet skin-contact service. Illumination, deformation-dependent optical coupling and composition-specific protection and safety therefore remain part of selecting a device for on-body use [37,38,39,40].

### 3.4. Conductive Polymers, Hydrogels, Ionic Conductors, and Nanofibers

Conductive polymers, hydrogels, ionogels, ionic conductors, and nanofiber networks address a recurring mismatch in wearable electronics: electrical function must coexist with skin-like mechanics. Poly(3,4-ethylenedioxythiophene):poly(styrene sulfonate) (PEDOT:PSS) and polyaniline (PANI) can be processed as fibres, coatings, composites, or electrochemical interfaces [31,45,46]. PEDOT:PSS fibre conductivity and mechanical response depend on formulation, microstructure, and processing [45,46].

Hydrogels support compliant ionic and conductive interfaces [47,48,49,50,51,52], while related formulations enable fibre energy storage [53]. Self-healing ionogel films serve as strain sensors, whereas ionogel coatings on textiles support physiological recording [54]. In a PEDOT:PSS hydrogel optoelectronic interface developed for neural and cardiac stimulation, Han et al. found that phosphate-buffered saline rehydration gave more stable photocurrent than deionized-water rehydration during 20,000 stimulation cycles in modified artificial cerebrospinal fluid [55]. The authors attributed this difference to reduced ion exchange. Separately, passive aging in artificial cerebrospinal fluid at 87 °C yielded 92 ± 2.8% photovoltage retention after 4.5 weeks [55]. These measurements characterize cyclic photocurrent and accelerated photovoltage stability, respectively, rather than observed multi-year implantation.

Fibre and yarn architectures shift this integration problem to the fabric, where loop and yarn geometry can influence the electromechanical response [56,57,58,59]. Coated and composite yarns, magnetoelastic fibres, nanofibrous webs and braided triboelectric structures offer different routes to deformable sensing [20,36,60,61,62,63,64,65,66,67]. Uzun et al. illustrate why the test object matters: MXene-coated cotton yarns retained MXene loading with little resistance increase after 45 wash cycles at 30–80 °C, whereas a separately assembled capacitive sensor using knitted MXene-coated bamboo-yarn electrodes was cycled 2000 times at 14.1% compression [20]. These distinct results support yarn washability and sensor cycling under their respective conditions. Garment-level measurement still depends on the assembled connections, fit and calibration after laundering.

### 3.5. Metallic Nanostructures and Liquid-Metal Composites

Metallic nanostructures serve distinct functions, from deformable, transparent Ag-nanowire conductors [68] to Au-nanobowl composites used for surface-enhanced Raman scattering (SERS) [69]. For an Ag-nanowire RF transponder embedded in a smart face mask, Ye et al. observed resistance increases of approximately 120% in uncoated conductors and 23% with PEDOT:PSS passivation after 30 days at 32 °C and 90% relative humidity [21]. The paired comparison shows how a protective layer affects conductor drift under a defined humid-storage condition. Stability at nanowire junctions and contacts under repeated deformation remains a distinct issue [70]; these resistance measurements do not themselves quantify RF detection after aging.

Liquid-metal composites present a related design problem: preserving conductive paths through deformation while controlling particle dispersion, oxide-shell activation, leakage and patterning [71]. Lee et al. printed polyelectrolyte-attached liquid-metal microgranular particles (PaLMPs) by meniscus-guided deposition, producing 50 µm lines on a substrate heated to 70 °C [72]. A printed PaLMP trace showed no electrical failure during more than 10,000 cycles at 100% strain; unencapsulated conductivity was reported stable over several weeks, and printed-line resistance was monitored separately for 30 days [72]. Importantly, thinner films showed larger strain-dependent resistance changes than thicker films. The authors exploited that difference in a prosthetic-hand e-skin, using thin serpentine sensing traces and thicker interconnects to reduce unwanted wiring response [72]. Printing geometry can therefore determine whether a conductor responds to deformation or preserves electrical continuity.

Plasmonic sensing requires a different kind of material tuning. Chung et al. used Au-coated thermoplastic-polyurethane (TPU) nanofibers for SERS sweat-pH sensing; selected Raman ratios yielded resolutions of 0.14 pH units (4-mercaptobenzoic acid; pH 5.5–8.0) and 0.51 pH units (4-mercaptopyridine; pH 4.0–8.0) [73]. Metallized nanofiber webs, by contrast, demonstrated stretchable transparent conduction, including integration into an organic solar cell [74]. These functional distinctions govern what needs to remain stable, what must change reproducibly and which chemical or optical reference is relevant for intended use.

### 3.6. Section Synthesis: Application-Driven Material Selection and Integration Trade-Offs

The material-selection problem is partly a question of where a response should occur. Layered MXene and MXene/CNT architectures operate across different compression and tensile ranges [4,34], while printed PaLMP traces use geometry to make one region strain-sensitive and another comparatively stable as an interconnect [72]. This contrast shows why device role and loading conditions are integral to material choice. Service evidence requires similar specificity. Ag-nanowire humidity drift and MXene-yarn washing assess different aspects of conductor and coating stability, whereas QD bending and electrolyte-conditioned hydrogel tests follow optical or optoelectronic outputs [20,21,44,55]. These results inform distinct operating conditions rather than a shared wearable lifetime.

Material-selection priorities therefore depend on the intended measurement. For motion and tactile sensing, calibration over task-specific loading and mounting conditions matters alongside sensor response [4,27]. Biochemical sensing requires selectivity and agreement with a suitable reference for the sampled medium [2]. Electrophysiological applications place different demands on skin contact and implanted insulation [3,5], while translating an optical detector to body-mounted use introduces additional light-coupling requirements [40,44]. Table 2 connects documented material-service and processing evidence (Part A) to conditional application-specific selection and verification priorities (Part B). Section 4 examines how electrode architecture, fabrication, packaging and contact with skin or tissue shape the integrated interface.

**Table 2:** Application-driven material selection from the reviewed evidence. Part A links representative material classes to device-specific service windows, aging and manufacturing constraints. Part B gives modality-specific test-first decisions. Both parts are author synthesis, not a validated standard, material ranking or class-wide lifetime assessment.

**Part A—Material-Specific Service Evidence**			
**Material Class/Route**	**Demonstrated Service Context**	**Limiting Mechanism/Process Constraint**	**Next Test and Evidence Boundary**
Carbon-based composites	Graphene/water–glycerol sensor: gauge factor ~2.4 at 25% strain; adhesion checked after ten stretches [27].	Network/contact changes and hydration; drop casting at 35 °C, copper/silver-epoxy terminals [27].	Recheck load/unload calibration, adhesion and intended exposure. Ten stretches do not establish prolonged wear.
MXenes and other two-dimensional materials	Packaged layered MXene sensor: 4000 compression cycles at 7.5 kPa [4].	Interlayer closure limits response range; film quality and PDMS/fabric package matter [4]. Oxidation remains a concern [29,30].	Match compression versus tensile loading; test wet drift and package integrity. Do not transfer this window to every MXene.
Perovskites and quantum dots	CsPbI_3_-QD/nanotube device: repeated deformation, with no unambiguous cycle-based window assigned [40]. Separate CsPbBr_3_-QD device: ~93% photoresponse-current retention after 1600 bending cycles; flexibility tested up to 60° [44].	Composition and barrier govern environmental/contact constraints [37,38,39]; film integration is device-specific.	Bending retention does not establish wet/sweat or prolonged human-contact lifetime. Assess optical drift, exposure/barrier stability and contact safety without assigning a class-wide lifetime.
Conductive polymers	Graphene/PEDOT:PSS electrode: 12 h EEG protocol including exercise and sweat [3].	Formulation, transfer and skin coupling; graphene growth precedes coating and transfer [3].	Test contact impedance, repeat attachment and motion noise. One volunteer protocol is not population-wide chronic qualification.
Hydrogels, ionogels, and ionic conductors	PEDOT:PSS hydrogel interface: 4.5 weeks of passive aging at 87 °C in artificial cerebrospinal fluid; 92 ± 2.8% photovoltage retention [55].	Phosphate-buffered saline rehydration preserved photocurrent better than deionized water during repeated stimulation [55].	Combine wet aging and cycling; verify the acceleration model before lifetime extrapolation. This window is not transferable to all gels.
Nanofibers and textile composites	MXene-coated cotton yarns: industrial-machine knitting and washing with retained loading and little resistance increase [20].	Coating and yarn/fabric construction constrain processing; geometry changes electromechanical response [19,20].	Check terminals, wash/cycle drift and re-donning after assembly. Yarn washing does not qualify an entire garment or factory yield.
Metallic nanostructures and liquid-metal composites	Ag nanowires: 30-day humidity storage gave ~120% resistance increase bare versus ~23% passivated [21]. Separate PaLMP film: >10,000 cycles at 100% strain without electrical failure; stable unencapsulated conductivity over several weeks, with resistance tracked for 30 days [72].	Ag-nanowire passivation/spray uniformity [21]; liquid-metal formulation/process constraints [71]. PaLMP meniscus-guided printing: 50 µm lines with a 70 °C substrate [72].	Test coupled exposure/deformation, containment/leakage and skin-facing contact barriers/safety. Do not transfer Ag-nanowire aging to liquid metal or assign a class-wide lifetime.
**Part B—Application-Driven Selection**			
**Modality/Operating Context**	**Selection Priorities and Test-First Sequence**	**Candidate Attributes/Verification**	**Caution/Evidence Boundary**
Motion/strain/tactile	First establish reversible, calibrated response over the task range; then test mounting and repeated-use variation [4,27].	Conditioned conductive networks or packaged layered sensors; load/unload, rate and exposure tests, followed by a task reference [4,19,27].	Peak gauge factor is not system accuracy; compressive and tensile windows differ [4]. Unseen-user evaluation is separate [9].
Biochemical/biofluid	First test selectivity, reference stability and temperature compensation; then sample renewal and enzyme/service drift [2].	Selective electrochemical interfaces with conditioning; interferents, shared-reference checks, fresh-sample comparison and in-use calibration [2].	In this array, refrigerated four-week storage and enzymatic-response degradation beyond the ~2 h exercise period are different endpoints [2]. Storage is not on-body service.
Electrophysiological	For skin, first test contact impedance/noise during motion; for implants, insulation and tissue response. Then, check longitudinal drift [3,5].	Conformal conductive films or insulated fibres selected for the site; signal/reference checks under motion/sweat, impedance tracking and tissue assessment [3,5].	The 12 h skin protocol and rat recording over months are different endpoints [3,5]; neither qualifies arbitrary prolonged human implantation.
Optical/optoelectronic	First test delivered/received light under coupling and deformation; then exposure-dependent optical/barrier stability [40,75].	Flexible photodetector stacks, hydrogel optoelectronic interfaces, or core–cladding fibres; evaluate optical transfer or photocurrent under deformation, wet/light aging, and contact-protection conditions [40,55,75].	Detector responsivity, optical delivery and stimulation endpoints are distinct. Mouse nerve modulation [75] is not prolonged human detector/safety validation.

Test-first order in Part B is conditional on the stated use; it does not rank failure probabilities. Abbreviations: EEG, electroencephalography; PaLMP, polyelectrolyte-attached liquid-metal microgranular particle; PDMS, polydimethylsiloxane; PEDOT:PSS, poly(3,4-ethylenedioxythiophene):poly(styrene sulfonate); QD, quantum dot.

## 4. Epidermal and Implantable Bioelectronic Interfaces: Architectures and Translational Constraints

### 4.1. Need for Soft and Conformal Bioelectronic Interfaces

Mechanical mismatch remains a central constraint in epidermal and implanted bioelectronics. Device contexts span neural recording and stimulation [76,77,78,79,80], optogenetic therapy using external goggles [81], tissue-compliant e-dura implants integrating neural electrodes and drug-delivery channels [82], and adaptive or conformal interfaces [83,84,85,86,87]. Although some implants achieve months of single-unit recording [5,88], poor skin contact can increase motion artifacts [3,89], whereas implant micromotion and foreign-body responses challenge chronic recording stability [87,88].

A usable bioelectronic interface must balance compliance, biocompatibility, long-term stability, and electrical or electrochemical contact under dry, wet, and dynamic conditions [88,89]. Substrate modulus and thickness are only part of the problem; a stiff or poorly conformal active electrode can negate a soft carrier. Nanomaterials offer small feature sizes, high surface area and adaptable interface structures [86,88,90]. Flexible and textile electrode studies show that electrode area, geometry, fabric construction and skin contact can affect electrodermal activity (EDA) and electrocardiography (ECG) signal quality [91,92,93,94,95,96]. Roll-to-roll Ag/AgCl yarn processing addresses manufacturability [97], while ambulatory electroencephalography (EEG) data quantify how head kinematics couple into motion artifact during physical activity [98].

### 4.2. Electrode Forms, Fabrication, and Device Architecture

**Figure 3:**
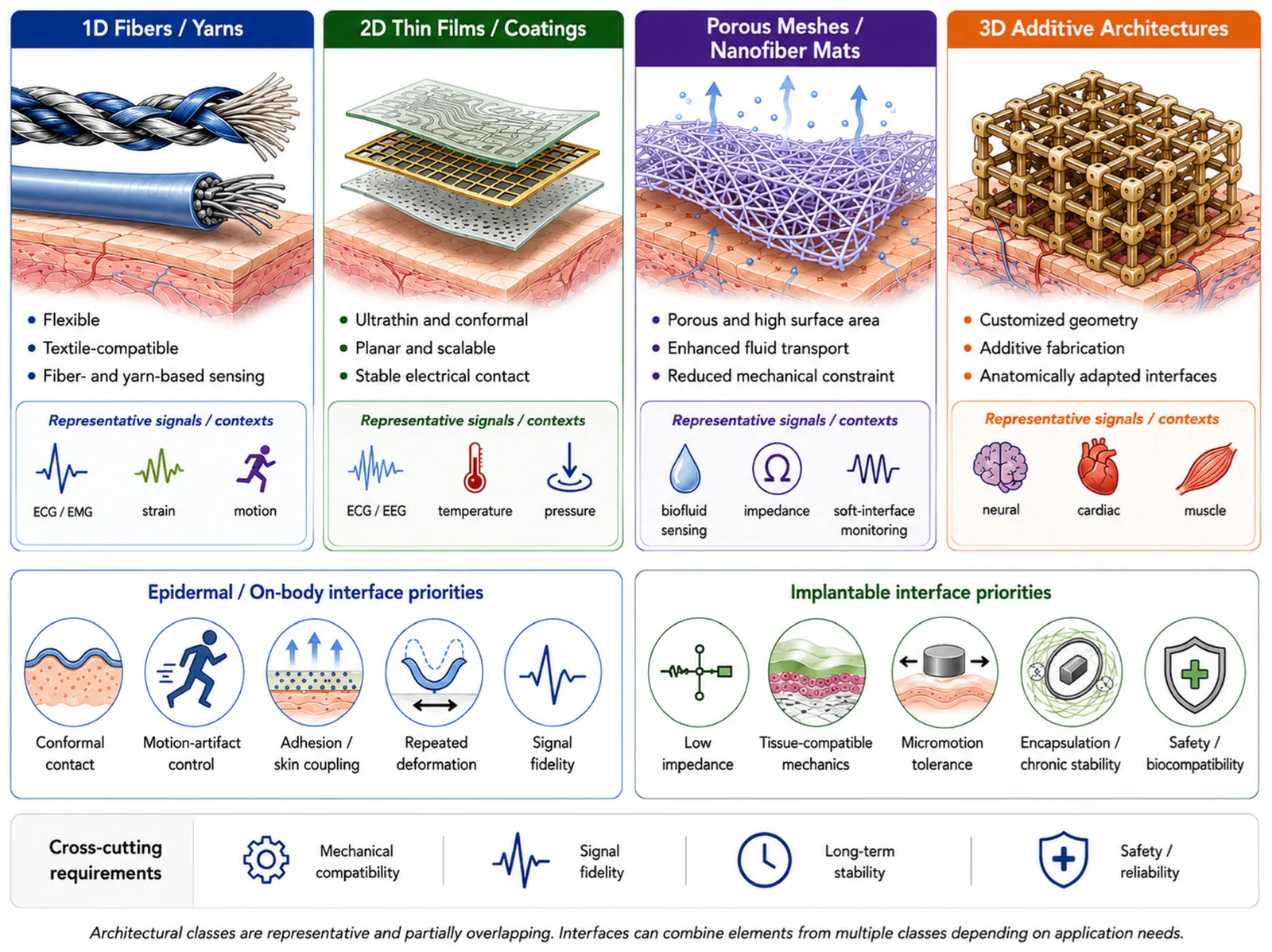
Representative bioelectronic interface architectures and design priorities. The upper panels summarize one-dimensional fibres/yarns, two-dimensional thin films/coatings, porous meshes/nanofiber mats, and three-dimensional additive architectures, together with representative sensing and interface contexts. The lower panels contrast key priorities for epidermal/on-body and implantable interfaces and highlight cross-cutting requirements in mechanical compatibility, signal fidelity, long-term stability, and safety. The architectural classes are representative and partially overlapping rather than mutually exclusive or sequential. Abbreviations: ECG, electrocardiography; EEG, electroencephalography; EMG, electromyography.

The conductive electrode couples bioelectronic interfaces to tissue for recording, electrochemical sensing and stimulation. Electrode architectures include one-dimensional fibres and yarns, two-dimensional films and planar arrays, porous meshes or nanofiber mats, and three-dimensional additive structures. Geometry connects conformability, active surface area and mechanical freedom with substrate compatibility, patterning resolution, unit operations, potential throughput and integration or encapsulation burden. Reproducibility must be assessed across the complete process, beyond electrode performance alone [3,97,99,100,101]. Figure 3 summarizes these design spaces for epidermal and implantable interfaces.

#### 4.2.1. One-Dimensional Fibres and Yarns

Fibres and yarns tolerate twisting and bending while retaining an extended conductive surface. Graphene/CNT and PEDOT:PSS routes include spun fibres and soft composites [45,46,102,103]; suitability for continuous manufacture depends on the formulation. In a laboratory roll-to-roll Ag/AgCl process on silver-coated nylon yarn, electrochemical coating at 2 mA and 0.13 cm s^−1^ was followed by rinsing, drying for 10 h at 60 °C and embroidery into fabric electrodes [97]. This connects continuous yarn coating to textile processing without planar-film transfer. Initial silver coverage, coating current, residence time and abrasion affect coating consistency [97]. Scale-up requires consistent coatings and downstream assembly. Process-cost drivers include feedstock, bath control, drying and fabric/contact assembly.

#### 4.2.2. Two-Dimensional Thin Films and Coatings

Ultrathin films and planar arrays provide conformal surfaces or defined recording sites, but their process requirements differ. Zhao et al. grew graphene on Cu at 1000 °C, spin-coated PEDOT:PSS, annealed at 120 °C, then etched Cu, rinsed and transferred the film to its final support [3]. Thermal processing precedes transfer; skin or flexible-carrier compatibility of the final device does not imply tolerance of the growth conditions. Driscoll et al. patterned Ti_3_C_2_ MXene on parylene-C using photolithography, sacrificial-layer dry lift-off, oxygen-plasma etching and evaporated masks, followed by parylene encapsulation and contact opening [99]. Aqueous MXene deposition therefore still requires microfabrication infrastructure in this route. Growth/deposition, lithography, transfer/release, alignment and encapsulation add process steps and equipment burden to conformal contacts or patterned arrays [3,99].

#### 4.2.3. Porous Meshes and Nanofiber Mats

Porous meshes trade continuous-film coverage for mechanical freedom and active surface area; on skin, open structures can improve breathability. Conductive components can be incorporated during spinning or added after fibre formation [62,63,64]. Electrospinning produces porous mats, but scale-up of the completed interface requires control of deposition uniformity, thickness/porosity, functionalization and contacts [62]. In a pressure-sensor example, silk nanofibers were collected on Cu, carbonized at 800 °C under Ar, transferred to PDMS after Cu dissolution and connected with silver paste/copper wires; a 45 × 45 mm membrane supported a patterned sensor matrix [101]. Collector compatibility, thermal processing, transfer and downstream integration must therefore be considered together with mat formation [62,101].

#### 4.2.4. Three-Dimensional Additive Architectures

Additive manufacturing enables electrode shapes beyond planar geometries. Oh et al. used pressure-driven direct ink writing of PEDOT:PSS–ionic-liquid colloids to produce approximately 50 µm features, suspended interconnects and vertical electrode arrays [100]. Ink preparation required freeze-drying, sonication and repeated centrifugation; rheology was adjusted for the intended geometry. This upstream processing remains necessary despite immediate electrode usability without additional post-treatment. Reported tool-path speeds were 6.4–12 mm s^−1^ for 2D patterns and 1.4–2.0 mm s^−1^ for omnidirectional 3D printing; these values do not measure completed-device throughput [100]. Implantable devices additionally used a PDMS support cured at 80 °C for 30 min and selective PDMS encapsulation, while skin tattoos used water-based transfer from a film [100]. Manufacturing these customized geometries includes material preparation, the print path, support, transfer and encapsulation.

#### 4.2.5. Manufacturing Compatibility and Scale-Up Trade-Offs

Manufacturability must be judged across the complete-device process, not deposition alone. Coated yarns can enter textile assembly but require reproducible coatings and contacts [97]. Transferred graphene films and patterned MXene arrays involve different thermal, transfer, lithographic and encapsulation steps [3,99], while porous mats and printed 3D electrodes introduce deposition-uniformity or ink-preparation requirements before integration [62,100,101]. These operations are identifiable process-cost drivers, not measured monetary costs. The examined studies provide no directly comparable factory yields or completed-device throughput: yarn length, membrane area, array layout and tool-path speed describe different production units. A suitable route therefore depends on required resolution, substrate compatibility, environmental protection and production volume, with repeatability verified after assembly.

### 4.3. Nanomaterial-Enabled Epidermal and Implantable Bioelectronics

Skin-contact evaluation depends on the signal being measured. A graphene/PEDOT:PSS film combined approximately 24 Ω sq^−1^ sheet resistance with a 12 h EEG protocol covering movement and sweating [3], providing material and on-body recording evidence. Carbonized silk membranes underwent more than 10,000 loading cycles with a reported pressure-sensor response below 16.7 ms [101], while battery-free patches measured pressure and temperature at multiple sites in patients [104]. StressFit combined electromyography (EMG) with sweat cortisol, pH and temperature sensing on functionalized laser-induced graphene during exercise and used pH and temperature to correct cortisol readings [105].

Biofluid sensing requires selective electrodes and reliable sweat delivery. Thread patches monitored Na^+^, NH_4_^+^, pH and lactate during exercise [106]; braided rGO/Pt yarns integrated lactate/pH sensing [107]. Pt/MXene–hydrogel microfluidics combined non-enzymatic glucose detection with sweat collection [108], while screen-printed textile electrodes reported glucose calibration and 30-day storage stability at 4 °C [109]. That storage endpoint differs from the on-body measurements in the yarn studies. Lee et al. coupled graphene/Au-mesh sweat glucose/pH sensing with thermally actuated metformin microneedles and reduced blood glucose in diabetic mice [110], demonstrating an additional preclinical therapeutic outcome. Conductive-hydrogel wound electrodes [111] provide adjacent stimulation-interface evidence.

Implantable neural electrodes must meet different requirements for recording quality, optical access and long-term stability. Patterned MXene microelectrodes had fourfold lower impedance than same-sized gold electrodes [99], while Au/PEDOT:PSS nanomeshes combined over 70% transmittance at 550 nm with simultaneous electrophysiology and two-photon imaging [112]. Pt-coated graphene-fibre electrodes recorded rat cortical activity with signal-to-noise ratios (SNRs) up to 9.2 dB [113]. Graphene/CNT fibres achieved conductivity of 3.3 × 10^5^ S m^−1^ [114], and support-free PEDOT:PSS fibres demonstrated chemical/electrical recording and stimulation [115]. These results address different functions rather than recording lifetime. In freely moving rats, CNT-fibre spinal electrodes with 2 µm parylene-C insulation yielded roughly two weeks of single-unit recording, versus up to 3–4 months with 4 µm [5]. Accompanying impedance changes implicated insulation leakage, linking longevity to the complete electrode rather than conductivity alone.

Optical biointerfaces differ in how light is coupled to neural or cellular responses. A curved MoS_2_–graphene retinal device used external electronics to convert photodetector output into electrical stimulation, eliciting visual-cortex responses in rats [116]. A PEDOT:PSS hydrogel (≈34 kPa) instead enabled capacitive photostimulation of cultured neurons and cardiac myocytes [55]. Reduced-graphene-oxide-clad poly(vinyl alcohol) (PVA) fibres delivered light to peripheral nerves in mice. Light-evoked muscle activity was assessed at six weeks post-implantation after locomotor testing, with EMG amplitudes followed through eight weeks; a separate experiment reported pain-hypersensitivity inhibition at eight weeks [75]. These results locate the critical engineering questions at different stages of the optical signal pathway: detector-to-electrode transfer, cell-interface charge delivery and light guidance during motion. Validation must therefore connect each stage to the observed biological response under the intended operating conditions.

An ocular accessory offers a passive link from monitoring to treatment. Cai et al. integrated microfluidic intraocular-pressure (IOP) sensing with pressure-gated timolol or brimonidine release in a battery-free, all-polymer contact lens [117]. Artificial-eye, ex vivo bovine-eye and rabbit ocular-hypertension studies reported pressure tracking and triggered delivery; rabbit studies compared IOP tracking with tonometry and reported IOP lowering similar to topical treatment in small groups, without establishing therapeutic equivalence [117]. Unlike the thermally actuated epidermal patch [110], treatment thresholds were encoded in channel geometry. The result demonstrates preclinical pressure-responsive intervention, while sustained use requires reliable pressure-to-dose coupling and ocular safety.

Within the gut–brain axis, chemical and bioelectrical signalling motivate different sensing interfaces [118]. NeuroString recorded evoked dopamine in mouse brain for up to 16 weeks, but measured gut serotonin acutely [119]. Conformable colonic electrodes separately recorded enteric electrophysiology in freely moving rats, including sessions up to two weeks after implantation in a two-rat pilot [120]. Together, these studies establish complementary preclinical capabilities across different sites and durations. Sustained multimodal gut sensing would require maintaining analyte selectivity and electrical contact under the same intestinal motion and biofluid exposure, with reference validation of both signals before stronger diagnostic or therapeutic claims.

### 4.4. Section Synthesis: Biointerface Stability and Translational Constraints

Section 4 shows that interface architecture shapes both fabrication and the function ultimately observed at the body. Textile contact geometry influences physiological recording, insulation affects neural recording longevity, and optical or microfluidic designs link light or pressure to biological responses. The reviewed fabrication routes and functional demonstrations establish complementary achievements, yet their process measures and validation endpoints differ in units, operating conditions and timescales. They therefore provide a limited basis for comparing manufacturing reproducibility with sustained functional performance across completed devices.

The key translational need is to connect these forms of evidence. Assembly and encapsulation consistency should be assessed alongside signal drift and function-specific reference measurements over the intended mechanical and biological service window, with sterilization and safety evaluated where relevant. Such evidence would clarify which limitations originate at the body interface and establish the signal stability and operating requirements that the readout, wireless and power architectures in Section 5 must accommodate.

## 5. Wireless Readout, Radio Frequency Sensing, and Power Strategies

**Figure 4:**
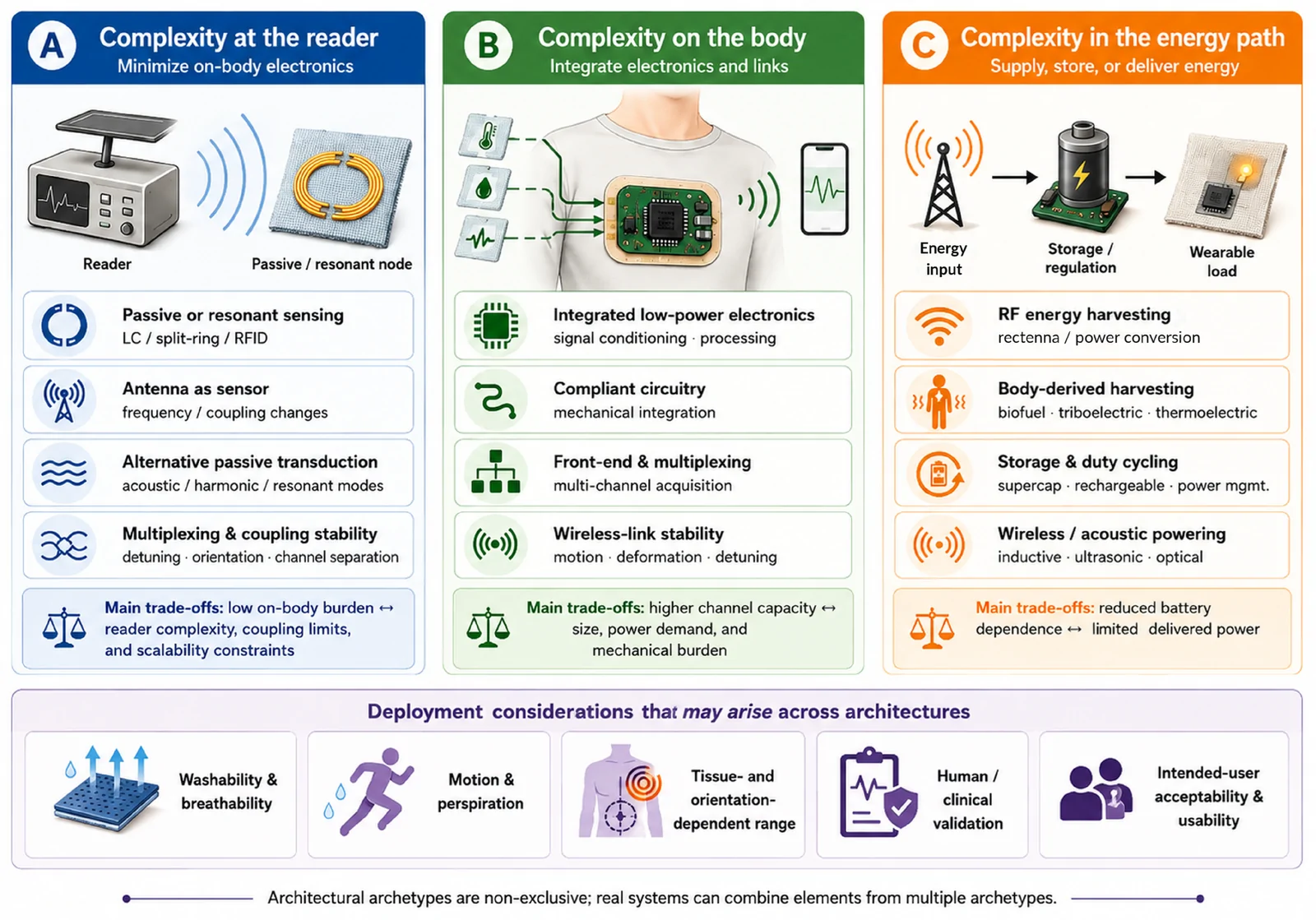
Wireless readout and powering architectures organized by where system complexity is concentrated. (**A**) Reader-centric approaches minimize on-body electronics through passive or resonant sensing; (**B**) on-body approaches integrate low-power electronics, multiplexing, and wireless links; and (**C**) energy-path approaches emphasize harvesting, storage, duty cycling, or wireless powering. Deployment considerations vary with architecture and use context, and the three archetypes are non-exclusive. Abbreviations: LC, inductor–capacitor; RF, radio frequency; RFID, radio frequency identification.

Beyond the biointerface, signal recovery and sustained operation depend on how readout, processing and power delivery are distributed. Section 5 compares reader-centric, on-body and energy-path architectures through reported link and load conditions (Figure 4; Table 3). Supplementary Table S2 provides study-specific operating and validation contexts for 35 records.

### 5.1. Passive and Resonant Radio Frequency Sensing

Passive inductor–capacitor (LC) interrogation places readout at an external reader, which tracks shifts in resonance, quality factor or backscatter caused by the sensing layer. A soft resistor–inductor–capacitor (RLC) bladder strain sensor based on gold-coated TiO_2_ nanowires in silicone had a modulus of 260 kPa at 40% strain (reported bladder tissue: 64–183 kPa). Readout persisted through 50% strain cycling at approximately 1 cm coil separation; several centimetres was stated only as an upper bound, and testing used ex vivo porcine bladder [121]. For oral chemistry, a silver-nanoparticle/chlorhexidine hydrogel split-ring sensor detected sulphide at 2–30 µM in artificial saliva (calculated detection limit: 1.2 µM) and released chlorhexidine in vitro. Saliva samples distinguished four periodontitis patients from three healthy volunteers [122]. An ultra-high-frequency (UHF) RFID antenna with a 110 nm PEDOT:PSS/graphene film converted temperature into received signal strength at 1.568 dBm/°C on a muscle-equivalent phantom. Gradient-boosted calibration trained on 35 of 50 participants yielded 0.437 °C mean error in 15 held-out participants [123]. These examples span ex vivo tissue deformation, oral-fluid analysis and calibration-dependent temperature estimation, each under different reader and reference conditions.

Nanomaterials can extend passive RF sensing beyond LC resonance by concentrating the sensing field or shifting the return frequency. A printed glucose-oxidase nanostrip microstrip approximately doubled sensitivity relative to a flat-film control across buffered glucose solutions (0.1 nM–10 mM; calculated detection limit, 0.1 nM). Bending altered its microwave response, and the on-skin test distinguished two simulated-sweat concentrations using a cabled vector network analyzer, not wireless readout [124]. A freestanding GaN surface acoustic wave (SAW) e-skin supported seven days of wrist-pulse monitoring in one wearer, alongside separate strain, ultraviolet and on-body sweat-Na^+^ demonstrations. Its near-field readout extended approximately 14 mm axially and 7.5 mm laterally, making reader alignment relevant to motion-tolerant operation [125]. Harmonic transponders instead separate the return frequency from the carrier. A graphene field-effect transistor (FET) showed a reported 1.38 V per pH Dirac-point shift in a biased-device test; the complete passive harmonic transponder was simulated rather than measured over a wireless link [126]. A Schottky-diode multiplier with silver-nanowire antennas on porous styrene–ethylene–butylene–styrene (SEBS) wirelessly tracked cough and mask displacement in one wearer over a fixed 0.5 m link [21]. The comparison places reader geometry and motion tolerance alongside sensor response as separate requirements for wearable RF design.

Passive RF arrays face spectral overlap as well as changes in coupling during deformation. Four tactile resonators using different multiwalled carbon-nanotube (MWCNT) loadings in PDMS were separated by 15 MHz, but their overlapping reflection-coefficient (S_11_) responses required a classifier. Held-out position accuracy reached 98.5%, with a pressure detection limit near 1 Pa [127]. A nonlinear parity–time (PT)-symmetric reader maintained coupling tolerance at reader–sensor separations below approximately 6 mm in its PT-symmetric phase, while a decoupling network reconstructed three-dimensional force from a four-channel MWCNT/PDMS array [128]. Patterned silver-nanofiber coils showed transmission efficiency falling from 35% to 15% at 10 MHz under 100% strain; near-field communication (NFC) and >1 m frequency-modulated links were demonstrated in separate tests [129]. A chip-free body-area network combined a CNT strain sensor (gauge factor 201.6 at 5% strain) with deliberately detuned tags that stayed readable across a resonance shift from 76.2 to 50.0 MHz under 50% strain. Vertical readout gain approached zero beyond 20 mm, whereas approximately 25 mm lateral antenna misalignment was tolerated; five tags monitored pulse, respiration and movement in two author-participants [130]. For on-skin sensing, a passive LC tag distinguished four forearm skin conditions in five participants through reflection-resonance measurements using an inductively coupled reader and an external vector network analyzer [131], while a battery-free NFC skin-moisture patch measured moisture through electrode resistance and examined antenna-size, bending and water-exposure effects on its wireless link [132]. Reliable wearable readout would require distinguishing sensing responses from changes introduced by deformation and reader coupling.

### 5.2. Readout Electronics and Wireless Links

On-body conditioning and telemetry offer an alternative to separating passive resonances at the reader, shifting constraints toward chip footprint and power. A batteryless, crystal-free system-on-chip (SoC) harvested RF energy into a flexible CNT supercapacitor. A 915 MHz broadcast supported carrier synthesis, while a process- and voltage-compensated on-chip clock eliminated the need for a quartz crystal. A 2.025 mm^2^ SoC consumed 24 µW per chip; a single-node test established a 457.5 MHz link over 1 m, while a separate two-node setup recorded arm EMG through fine-wire electrodes [133]. A 4 × 4 mm SoC fed by CNT-fibre electrodes integrated signal conditioning, power delivery, communication and power management in a sweat patch. With two off-chip passives, it drew a minimum average of 33 µW at a 1:60 RF duty cycle (analog measured, digital simulated). The patch monitored potassium and lactate during exercise in one volunteer [134]. These demonstrations show low-power on-body readout under distinct sensing workloads and duty-cycle conditions.

Compact SoCs leave flexibility and channel density as separate readout constraints. Stretchable polymer diodes rectified 13.56 MHz RF signals and enabled an RFID tag integrating a sensor, electrochromic pixel and antenna [135]. A flexible, coin-cell-powered front end coupled CNT-FETs bearing a nonactin-based ion-selective membrane to a Bluetooth SoC for ammonium sensing in artificial sweat (0.1–100 mM; reported sensitivity 4.516 µA/decade; five devices). Representative calibrations agreed with a benchtop analyzer at feedback resistances of 10 and 39 kΩ, whereas the readout saturated at 82 kΩ for four of five devices [136]. A wafer-scale trilayer MoS_2_ array on polyimide reached 51 pixels mm^−2^ with a reported approximately 20 ns on-site multiplexing time constant; electrocorticography was tested in vitro and in living mice [137]. The appropriate circuit architecture therefore depends on whether RF rectification, chemical-readout range or dense multiplexing constrains the intended task.

Skin deformation can detune RF components and weaken wireless links. A dielectro-elastic substrate limited strain-induced resonance shifts to ~5 MHz, compared with 50–1250 MHz reported for other elastomer-supported antenna designs; skin-interfaced telemetry reached up to 30 m under tested deformations [6]. Embroidered near-field clothing extended power and data connectivity among battery-free sensors at different body locations [138]. At network scale, simulation-based optimization jointly considered relay placement and transmission power to improve network lifetime [139].

### 5.3. Power and Energy-Harvesting Strategies

Wireless readout does not remove the on-body energy requirement. Three strategies recur in the Section 5 literature. 

The first harvests RF energy. An atomically thin MoS_2_ semiconducting-metallic heterojunction rectifier reached a 10 GHz cutoff and, with a flexible antenna, rectified 5.9 GHz radiation at zero external bias. It delivered up to 250 mV at approximately 2.5 cm from a signal-generator-driven antenna with roughly 2 mW available at the rectenna; true ambient harvesting remained future work [140]. A fully textile 5.8 GHz receiver in a glove powered yarn bend sensors at distances up to 1 m from a 30 dBm laboratory source and horn antenna. The ratiometric signal remained stable as rectified voltage fell with distance, and the system classified grasped objects at 98.75% [141].

A second strategy draws on biochemical, mechanical and thermal energy conversion, alongside acoustic power transfer for potential implants. A lactate biofuel-cell e-skin reached 3.6 mW cm^−2^ in ex vivo sweat from four donors (3.5 mW cm^−2^ in the abstract); a separate approximately 60 h charge–discharge test involved fuel replenishment in vitro. On-body sensing with duty-cycled Bluetooth was demonstrated separately in three volunteers [7]. A 3D-printed MXene triboelectric generator produced an instantaneous peak of 816.6 mW m^−2^ into 100 MΩ under motor actuation (8 Hz, 19.9 N). Pulse monitoring in one volunteer depended on recharging a storage capacitor [142]. A five-leg Ag_2_Se thermoelectric generator delivered 4.25 µW at ΔT = 35 K under matched loading; a separate hot-water demonstration required voltage boosting from approximately 20 mV. The results report 85 W m^−2^ normalized to total thermoelectric-leg cross-sectional area, while the abstract and conclusion quote 81 W m^−2^; this difference remains unresolved [143]. A CsPbI_2_Br/poly(vinylidene fluoride) piezoelectric yarn yielded a peak of 281.0 µW cm^−3^ under 16 kPa actuation into 300 MΩ, normalized to nanofiber-layer volume. It charged a 1.1 µF capacitor to 1.8 V in 50 s, with the stored charge used to light an LED rather than operate a sensing node [144]. An externally driven ultrasonic device produced 1.2 V and 150 µA through 10 mm of excised porcine tissue and supported passive wireless sensing in separate tests [145]. For deployment, the relevant question is how much energy reaches the sensing and communication workload under realistic input and recharge conditions.

The third strategy manages reader-dependent or intermittent power by coordinating sensing with communication and local storage. A battery-free epidermal NFC platform required a nearby smartphone; in three volunteers, sweat-derived blood-glucose predictions using 120-fold scaling had a reported mean absolute relative difference (MARD) of 13.8% against fingertip measurements, while uric acid was assessed separately [146]. Where continuous reader access is impractical, a fully 3D-printed microsystem combined wireless charging, sodium-ion microbatteries and glucose-sensing. The thickest battery stack reached 0.96 mAh cm^−2^; a thinner cell recovered 34.6% of receiving-coil output energy on discharge, while the integrated system detected glucose down to 0.5 mM in simulated sweat [147]. A zinc-ion fibre battery with a graphene-oxide/poly(vinyl alcohol) electrolyte powered on-body multimodal textile monitoring; cycling beyond 500 h and 98% capacity retention after 1000 cycles at 0.3 A g^−1^ were reported in separate cell-level tests [53]. These architectures therefore call for distinct deployment checks of reader proximity, recharge requirements and sustained delivery under the intended sensing load.

### 5.4. Integration, Robustness, and Deployment

Washability and motion probe different aspects of textile-system robustness. Silk-fibroin coils incorporating carbon nanotubes and chitin-derived carbon nanoflakes supported wireless operation after 50 detergent-free machine-wash cycles; silanization imparted omniphobicity while retaining breathability [148]. A waterproof triboelectric pulse sensor reported a 23.3 dB signal-to-noise ratio and a 40 ms bench response, with pulse monitoring during movement and moisture exposure demonstrated qualitatively. In one participant, blood-pressure estimates showed reported mean relative deviations of 2.9% for systolic and 1.2% for diastolic pressure against a commercial cuff [149]. These demonstrations distinguish physical resilience from the narrower evidence available for validating inferred physiological measurements under use.

Deployment evidence extends short-term robustness tests by revealing how performance changes with wear, reader coupling and evaluation protocols. For silver-nanowire ECG electrodes, blinded assessment of 12-lead recordings from 20 volunteers found no discernible differences in evaluated ECG features relative to commercial electrodes. Yet normalized R-peak values declined by about 35% for both electrode types at 24 h, and the printed layer detached after separate 24 h artificial-sweat exposure. The quoted 142 m wireless range was calculated, not measured [150]. A phone-powered wound bandage demonstrated iontophoretic delivery in vitro and accelerated healing in infected diabetic rats, while sensing was evaluated separately in 10 patients, six followed for two weeks. Its reported glucose, pH and temperature accuracy scores against commercial references exceeded 80% across 25 plotted measurement–reference pairs per output [151]. Passive stent sensors tested in an artery phantom and ex vivo ovine coronary arteries showed radial/axial read ranges falling from 2.5/1.0 cm in air to 1.5/0.5 cm in blood-conductivity-matched saline, with signal loss at 90° antenna rotation [152]. A phone-powered throat patch operated over 3–4 cm and yielded 79.4–81.6% text accuracy in four-participant leave-one-subject-out testing, distinct from separately reported finalized-model accuracies of 84.71% for text and 90.20% for emotion [153]. The next validation step should therefore target the relevant failure mechanism—signal degradation, wireless coupling or transfer across users and settings—using tests matched to the intended claim.

**Table 3:** Architecture-level synthesis of wireless readout, radio frequency sensing, power, and deployment evidence in Section 5. Representative examples are shown; the detailed 35-study Section 5 evidence table is provided in Supplementary Table S2.

Strategy/Evidence Domain	Representative Evidence and Demonstrated Capability	Principal Integration and Deployment Constraints
Fully passive and resonant RF sensing	Battery-free or chipless resonant sensing has been demonstrated for mechanical, temperature, biochemical, and tactile measurements. Representative examples include an RLC sensor tested ex vivo on porcine bladder [121], UHF RFID temperature sensing with held-out human validation [123], and a multi-resonator tactile array with learned decoding [127].	Reader–sensor coupling depends on distance, orientation, surrounding medium, and deformation. Multisensor scaling can cause resonant-peak crowding or overlap, and some systems require model-based decoding; usable range may also remain limited under realistic coupling conditions.
On-body readout electronics and wireless links	Integrated approaches include a batteryless low-power SoC supporting synchronized EMG acquisition [133], high-density MoS_2_ active arrays with rapid multiplexed readout [137], and strain-compensated RF electronics designed to preserve wireless operation during deformation [6].	Rigid electronics can increase mechanical burden, while front-end range and saturation, external-controller dependence, power budget, multiplexing requirements, and deformation-induced RF detuning can limit integrated performance.
Power, energy harvesting, storage, and charging	Representative strategies include RF energy harvesting [140], body-derived biofuel-cell power supporting on-body wireless sensing [7], battery-free NFC-enabled sweat sensing [146], and wirelessly charged stretchable microsystems with integrated energy storage [147].	Available energy may be intermittent, and laboratory excitation does not establish autonomous operation under ambient or body-derived conditions. Peak output can differ substantially from sustained power delivered to the actual sensing, processing, and communication load; storage, voltage conversion, duty cycling, and recharge requirements add further system constraints.
Deployment robustness and human/clinical evaluation	Deployment-oriented evidence includes washable textile receiving coils [148], blinded clinician assessment of wearable ECG recordings [150], preliminary patient evaluation of a wireless wound-sensing platform [151], body-medium- and orientation-dependent implant readout [152], and subject-independent evaluation of a battery-free throat interface [153].	Washability, perspiration, movement, tissue attenuation, antenna alignment, long-duration failure, small or heterogeneous cohorts, and user-to-user generalization can become limiting once systems move beyond benchtop integration.

Abbreviations: ECG, electrocardiography; EMG, electromyography; NFC, near-field communication; RF, radio frequency; RFID, radio frequency identification; RLC, resistor–inductor–capacitor; SoC, system-on-chip; UHF, ultra-high frequency.

### 5.5. Section Synthesis: Readout, Power, and Deployment Trade-Offs

Nanomaterials now contribute to readout and power as well as transduction, shifting the focus from isolated component outputs to the usable signal path. Passive resonators reduce on-body power demand but depend on reader coupling and resolvable channels; active electronics enable conditioning and multiplexing at a power and integration cost. Harvesting and storage support operation only when the energy supplied can sustain sensing, processing and communication together.

These choices become interdependent during wear, as body motion can affect both sensor contact and wireless coupling, while environmental exposure challenges repeatability. The reviewed on-body and clinical demonstrations establish feasibility, but their differing durations, references and participant populations limit comparisons of sustained performance. Section 6 therefore examines whether an assembled platform preserves its intended measurement when sensing, readout and power operate together under the conditions of use.

## 6. Integrated Wearable Platforms for Human Motion, Functional Monitoring, and Tactile Interaction

Acquiring a stable signal is only the first step in establishing what a wearable platform measures. Form factor and placement influence what is sensed directly and what must be inferred, which in turn determines the appropriate calibration and reference measurement. Section 6 examines how these relationships shape the validity of movement, functional-monitoring and tactile platforms, beyond isolated component sensitivity or classifier accuracy.

### 6.1. Wearable-Platform Taxonomy for Human-Centred Sensing

**Figure 5:**
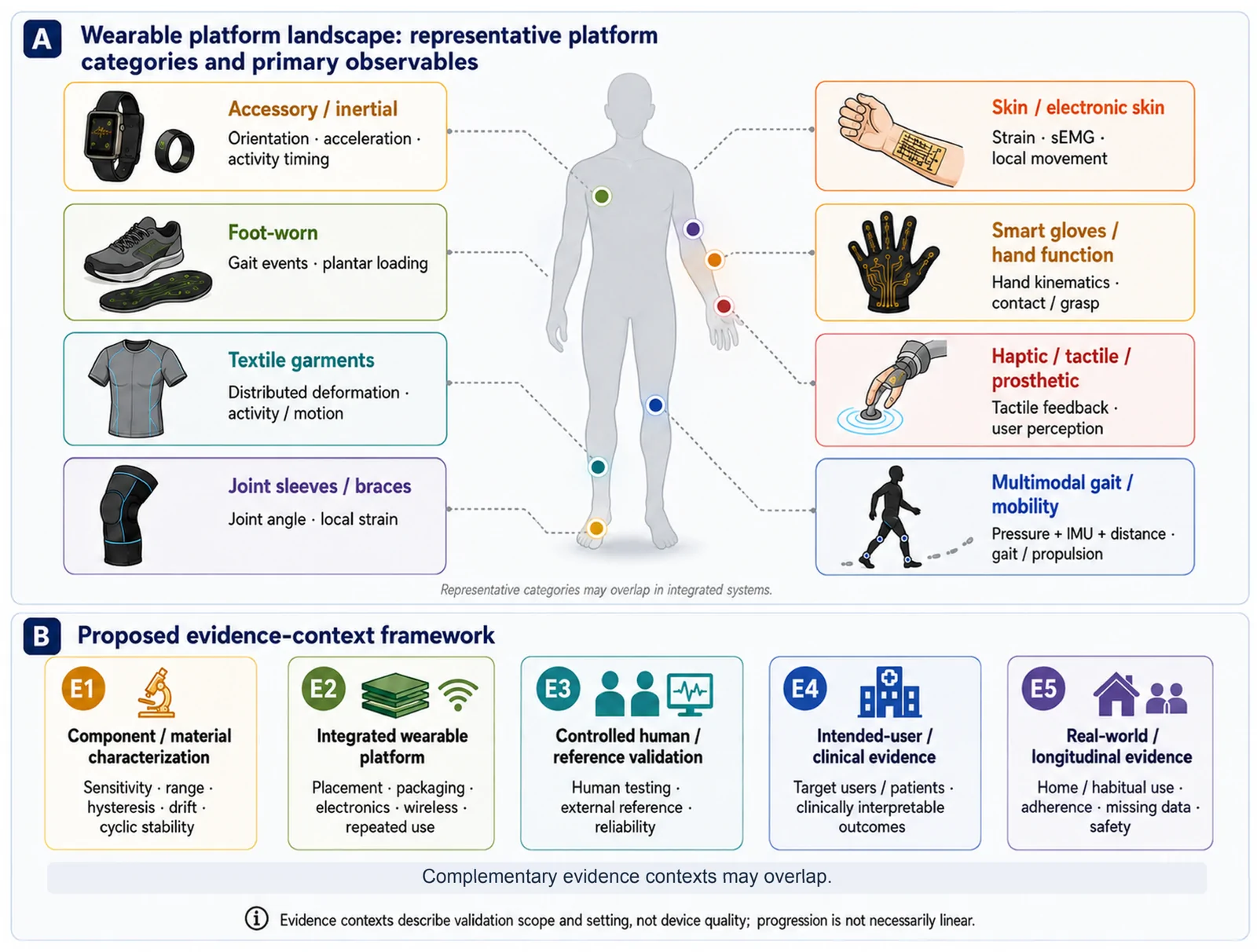
Wearable-platform landscape and proposed evidence-context framework. (**A**) Representative wearable-platform categories and their primary observables, spanning inertial, foot-worn, textile, joint-mounted, skin-interfaced, hand-function, haptic, and multimodal mobility systems. (**B**) Five complementary evidence contexts from component/material characterization to integrated wearable testing, controlled human/reference validation, intended-user/clinical evidence, and real-world/longitudinal evidence. The contexts address different evidence questions, may overlap, and do not indicate device quality or a regulatory sequence; progression is neither mandatory nor necessarily linear. Abbreviations: IMU, inertial measurement unit; sEMG, surface electromyography.

We group wearable platforms by form factor and observable: accessory and inertial systems, foot-worn devices, textile garments, joint sleeves and braces, skin-mounted systems, smart gloves, haptic or prosthetic interfaces, and multimodal mobility platforms (Figure 5). Categories overlap and imply no hierarchy: gloves can combine motion sensing with feedback, and gait platforms can combine pressure, inertial and ranging signals. Each form factor observes different variables and fails differently.

Inertial measurement units (IMUs) provide acceleration and angular-velocity signals from which segment orientation and activity timing can be estimated. Foot-worn inertial sensors help identify gait events, whereas pressure-sensing insoles measure plantar loading; garments and sleeves capture distributed textile deformation or local joint strain. Skin interfaces acquire strain, surface electromyography (sEMG) and other physiological signals. Smart gloves can acquire finger-deformation or contact cues to estimate hand motion and grasp, while haptic and prosthetic systems add actuation or sensory feedback. Multimodal combinations relate kinematics, kinetics, neuromuscular activity and functional performance.

Evidence roles follow the classification in Section 2.3. Conventional electronic platforms serve as comparators for integration, reference validation, repeated-use and deployment requirements that nanomaterial wearables must also meet. Systems with a nanomaterial or nanostructured component in the sensing or interface path are treated as direct evidence. Mixed platforms are classified by their role in the synthesis.

### 6.2. Gross-Movement, Gait, and Foot-Worn Sensing Platforms

Accessory and inertial wearables estimate exercise dose, segment motion, joint kinematics, and spatiotemporal gait variables. Full-body and compact inertial systems have been compared with optical motion capture and electronic walkways during athletic and gait tasks [154,155], and smartwatch platforms have supported home exercise in people with chronic stroke [156]. For loading or contact inferred from motion, output error depends on sensor placement, calibration, and anatomical alignment.

Patel et al. separate laboratory validity from outdoor feasibility. Fifteen participants without reported disabilities completed the study, and the eight assessed outdoors were drawn from the same indoor cohort. Indoor measurements were collected concurrently with Vicon; the outdoor comparison was recorded at a different time [8]. The data therefore establish laboratory reference agreement and outdoor operability, but not concurrent outdoor accuracy or validation in a patient population.

Foot-worn platforms can characterize gait through inertial sensing and directly measure plantar loading when pressure sensors are included. Smart insoles and shoes have been compared with optical references [157], tested for simulated shuffling in healthy participants [158], and evaluated in people with Parkinson’s disease and matched controls [159]. An e-textile sock was tested in nine healthy participants, including six men, and established repeatability across walking speeds rather than patient-population validity [160]. Footwear, surface, sensor coverage, gait speed, calibration drift, and the definition of the reported outcome remain major error sources.

Multimodal gait systems combine pressure, inertial, and ranging signals to connect movement with loading. The pressure/inertial/distance-sensing platform was tested in healthy participants and people with gait disorders under structured laboratory protocols and later examined during habitual activity [161]. Swaminathan et al. studied 13 unique participants in the main study—six after stroke and seven unimpaired—with one repeat visit yielding seven clinical datasets. Separate demonstrations reused participants and added two post-stroke participants, giving 15 unique participants overall [162]. Force plates supplied laboratory ground truth for propulsion, whereas out-of-laboratory measurements established feasibility without concurrent reference data. Complementary sensing therefore broadens the observable set, but synchronization, personalization, sensor failure, and real-world ground truth remain unresolved. Few-shot learning with generative augmentation for pathological-gait forecasting addresses a related limitation: sparse labelled data across users [163].

### 6.3. Textile Garments, Sleeves, Braces, and Skin Interfaces

Textile garments embed sensing into a structure that already contacts the body, allowing distributed measurements without multiple rigid modules. The signal is not purely anatomical. Fabric mechanics, garment tension, body morphology, slippage, and re-application are relevant measurement conditions [19,164]; the sensor-yarn glove study considered response and fit variations across participants or sessions [165]. Optical-fibre smart pants demonstrated activity classification under a defined protocol, without establishing cross-user generalization [166]. A seamlessly knitted multisensor garment was compared with Vicon during a pick-and-hold task in one healthy participant; goniometry defined static scanning poses [164]. Other examples include knitted respiratory sensing [167,168] and a seatbelt platform that combines ECG, respiration, motion sensing, and wireless transmission [169].

Joint sleeves and braces constrain sensor placement around a known anatomical region, which is useful for range-of-motion, joint-angle, exercise-form, and feedback measurements. A knitted shape-memory-alloy elbow brace was tested in ten healthy participants for heat, compression, blood flow, and range of motion; it was not evaluated for clinical efficacy in osteoarthritis [170]. A graphene knitted sleeve was evaluated for angle estimation, cross-participant testing and gesture classification; durability, environmental aging and washing were assessed on constituent sensors [171]. By contrast, the carbon-nanocomposite elbow sleeve and KneeSense brace primarily establish system integration because intended-user evaluation was limited or not clearly reported and controlled clinical validation was absent [172,173]. A smart-textile knee sleeve mapped wearable signals to 3D lower-body kinematics and was evaluated against motion capture, including unseen activities and users [174].

Hydrogel and electronic-skin interfaces couple tightly to the body and acquire strain or sEMG signals to estimate motion and posture, monitor fatigue-related changes, and support human–machine interaction. Zhang et al. compared participant-averaged strain-signal and optical joint-kinematic curves from nine healthy participants; the participant count for separate full-day demonstrations was not clearly reported [175]. Yang et al. reported three participants for neck movement, three for fatigue monitoring, and two for an alert demonstration, but overlap between groups and the number of unique participants were unclear [176]. Fit, hysteresis, washability, adhesion, sweat, biofouling, and session-to-session calibration remain practical barriers across garments, sleeves, braces, and electronic skins.

### 6.4. Smart Gloves, Tactile Interfaces, and Haptic Systems

Hand-worn systems serve different endpoints: kinematic measurement, clinical assessment, rehabilitation, gesture interaction, prosthetic feedback, and sensory substitution. The validation target must match the claim. Optical tracking can establish kinematic accuracy; claims of functional or clinical benefit require relevant outcomes assessed with appropriate validated measures. Gesture-recognition accuracy alone establishes neither.

Stroke studies include a prematurely terminated multicenter smart-glove trial, a completed randomized controlled trial, and a small randomized home study [177,178,179]. Together they establish feasibility and report functional outcomes, but cohort size, intervention duration, adherence, and generalizability limit interpretation. A multimodal assessment glove compared flexibility and strength grades with clinician ratings in 12 participants (four with Parkinson’s disease); a separate tremor assessment involved 32 healthy participants simulating tremor and eight patients with Parkinson’s disease [180].

Engineering prototypes answer narrower questions. Li et al. compared hand tracking with an optical reference, but the total number of participants was not clearly stated and no evaluation in participants with cerebral palsy was reported [181]. A flexible optical-fibre glove recognized gestures and movements in four participants, constraining the reported classification result to that prototype dataset [182]. A controlled glove simulator is useful for repeatable component tests but does not constitute human validation or clinical evidence [183].

Haptic and prosthetic platforms add actuation and perception to the signal chain. Bruni et al. tested stiffness recognition and vibrotactile feedback in 18 participants without amputation and five amputees, directly sampling the intended prosthetic-user population [10]. GaitSkin was tested in five healthy participants and establishes preliminary timing feasibility for gait-linked haptic stimulation, not Parkinson’s disease or clinical gait validity [184]. A multimodal nanomembrane glove combined pressure, temperature, and flexion sensing with feedback in healthy volunteers, but the total participant count was not clearly reported and children with traumatic spinal cord injury were not included [185]. Repeated-use studies still need to quantify feedback safety, comprehension, acceptance, and functional benefit.

### 6.5. Proposed Evidence-Context Framework for Translational Evaluation

We use five complementary evidence contexts (E1–E5) to distinguish the claims supported by wearable studies (Figure 5). E1 characterizes material or component performance under specified physical, environmental, and test conditions. E2 characterizes integrated wearable performance after packaging, placement and body coupling, including electronics, power, wireless and complete-device operation, and repeated-use where relevant. E3 provides controlled human evidence for accuracy or agreement against an appropriate external reference, or repeatability against a predefined reliability protocol; reliability alone does not establish accuracy. E4 provides interpretable, usable evidence in the intended population and intended-use context, without equating participation with clinical usefulness or benefit. E5 addresses performance, usability, safety, adherence, data completeness, and operational stability during everyday and/or longitudinal use. Real-world setting and longitudinal duration remain separate attributes.

A study may support multiple overlapping contexts, with neither mandatory nor linear progression. E1–E5 have not been validated as a quality scale and convey no ordinal ranking, maturity ladder, or regulatory pathway. E5 is not inherently stronger than E3 or E4; relevance depends on the claim. Existing standards and guidance address related questions in risk management [186], basic safety and essential performance [187], usability engineering [188], clinical investigation [189], and remote data acquisition [190], but do not define E1–E5 as a consensus medical-device framework or prescribe them as a unified sequence. Supplementary Table S6 provides the corresponding standards/guidance crosswalk. Cross-platform accuracy comparisons require aligned tasks, populations, references, and settings. Table 4 summarizes platform validation contexts and limitations.

### 6.6. Platform-Level Evidence and Translational Implications

Platform validity follows from how each device maps a sensor signal to the quantity being claimed. Inertial estimates of loading rely on an inferred motion–load relationship, whereas plantar pressure depends on contact at the foot–insole interface. Garment fit and re-donning can change textile–body coupling, while glove and haptic systems must be judged against their intended tasks and, where relevant, feedback responses. Calibration, reference selection and repeated-use testing must therefore follow the complete measurement pathway rather than component performance alone.

Those form-dependent relationships also determine how far controlled results can be transferred. Small or healthy cohorts and non-equivalent references constrain conclusions for intended users; re-donning or calibration drift can change the signal–output mapping even when a device continues recording. Technical failure and user burden further affect data availability, while concurrent real-world reference evidence remains uneven. Section 7 therefore considers whether changing inputs can support reliable estimates and feedback, and Section 9 addresses how that evidence should be reported.

## 7. AI Inference/Interpretation, Multimodal Sensor Fusion, and Feedback

The functional outputs considered here often have to be inferred from sensor signals. A resistance trace from a knitted strain sensor is not a joint angle, and a tactile voltage map is not a grasp force. The interpretation layer must transform those raw signals while remaining constrained by the mechanics, drift, noise, and placement sensitivity of the transducer. This systems problem links the material and interface behaviour described in the preceding sections to the model outputs considered here.

We organize machine learning by the sensor problem it is asked to solve, not by model family. Temporal memory, channel weighting, transfer learning, multimodal fusion, and uncertainty estimation are examined as responses to specific imperfections in soft sensing. Model choice is therefore evaluated against sensor behaviour and the validation needed to support the resulting claim. Table 5 summarizes those links.

Motion, tactile, neuromuscular and multimodal examples make calibration, generalization, robustness and feedback constraints concrete; selection limits are discussed in Section 10.5.

### 7.1. Signal Preprocessing, Calibration, and Feature Extraction

Preprocessing is part of the sensing system, not a neutral software step. Deformation history, hysteresis, skin contact and environmental exposure can alter soft-sensor signals [19,27]. Filtering, baseline correction, hysteresis compensation, normalization, segmentation and channel synchronization therefore encode sensor- and task-specific assumptions that may not transfer across wear conditions.

Calibration must also be practical beyond laboratory conditions. Self-supervised tactile calibration improved reference-signal correlations from 77.7% to 88.3% (glove versus digital scale) and from 32.1% to 74.2% (vest versus calibrated glove) [191]. Robotic tactile studies further demonstrate transfer across physically distinct sensors [192,193]. In an MXene textile glove, local near-sensor updates raised 20-class gesture accuracy from 70.5% to 98.1% after expansion from 14 classes [194]. However, a crack-based wrist-sensing system required additional data and retraining for new users or placements [195]. Together, these studies show that learned calibration can reduce—but not eliminate—device- and use-specific calibration burdens. The subsection “Generalization, Personalization, and Calibration Burdens” in Section 7.3 identifies the corresponding reporting requirements.

### 7.2. Machine Learning for Motion, Force, Posture, and Intent Estimation

The selected tactile and textile studies illustrate distinct outputs, including discrete recognition and continuous estimates of motion or force. Early tactile gloves captured enough spatial information for object identification and analysis of human grasp structure [196], and the same concept scaled to conformal textiles covering larger body regions [191]. Later systems reconstructed articulated hand kinematics from a washable sensor-yarn glove under within-participant and between-participant evaluation [165], or estimated distributed force fields during interaction with deformable objects [197]. Smart-textile knee sleeves provide a wearable lower-limb counterpart by mapping distributed textile deformation and inertial measurements to continuous joint-angle outputs [174].

**Figure 6:**
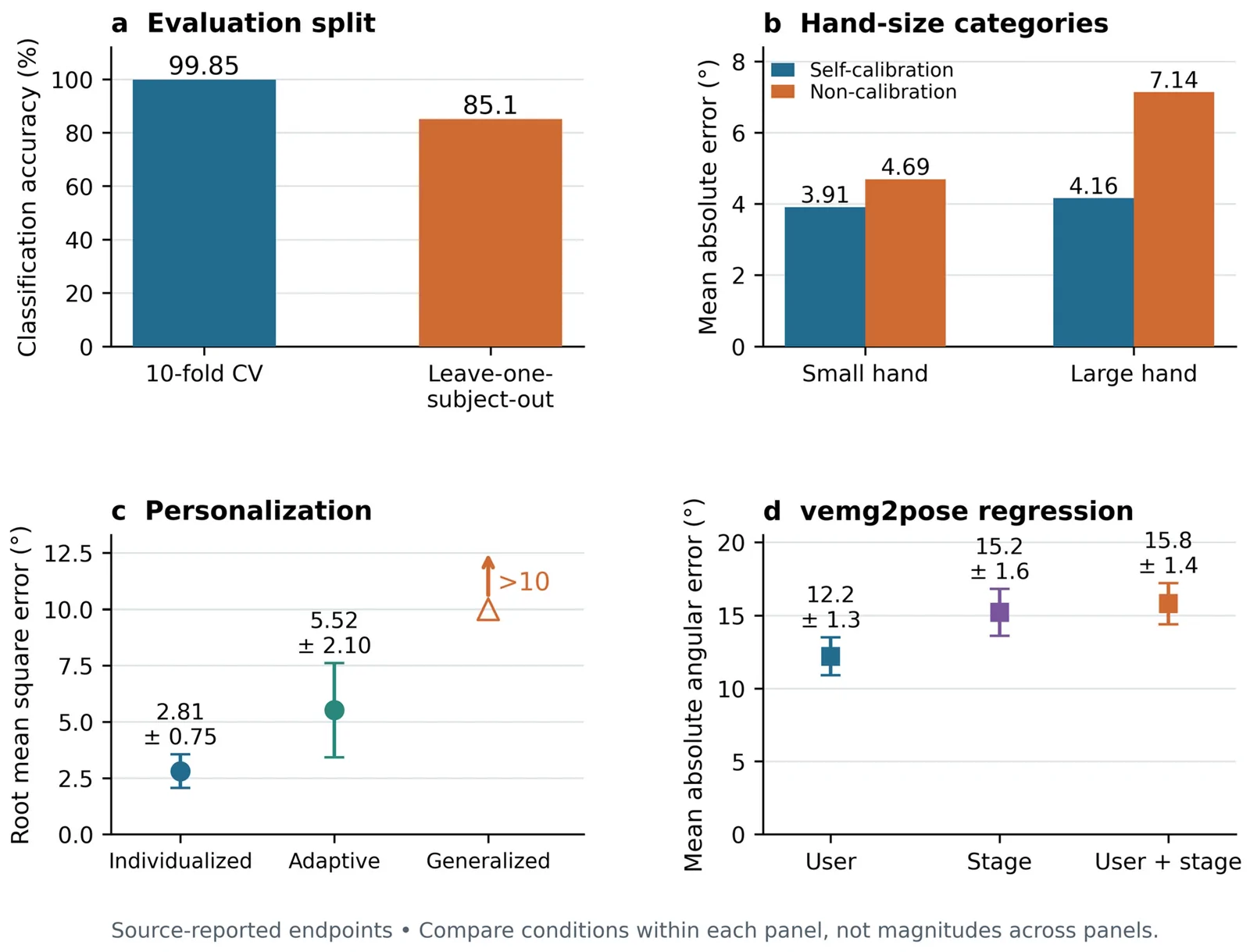
Evaluation conditions and source-reported within-study performance. (**a**) Reported classification accuracy for the same convolutional-network configuration under 10-fold cross-validation (99.85%) and leave-one-subject-out evaluation (85.1%) [9]; the observed 14.75-percentage-point gap is not an isolated causal effect of leakage. (**b**) Mean absolute joint-angle error for distinct self-calibration and non-calibration participants: 3.91° and 4.69° for small hands, and 4.16° and 7.14° for large hands, respectively [201]. Each bar averages ten joint degrees of freedom for one participant; there is one participant per hand-size/calibration cell and no across-participant uncertainty estimate. (**c**) Long short-term memory model root mean square error (RMSE) under individualized (2.81 ± 0.75°), adaptive (5.52 ± 2.10°) and generalized (>10°) training [22]. Error bars denote one standard deviation across joint-angle outputs in the common single-participant last-20% test segment. The open triangle and upward arrow mark a strict lower bound, not an estimate of the generalized mean. (**d**) vemg2pose regression mean absolute angular error and one standard deviation across users [204]: held-out users on stages represented in training (12.2 ± 1.3°), held-out stages performed by users represented in training (15.2 ± 1.6°), and held-out users performing held-out stages (15.8 ± 1.4°). Stages are categories of hand movements. These are distinct test sets, not a paired deterioration experiment; tracking with a known initial pose is a separate task. Values are not normalized, and magnitudes must not be ranked across studies because tasks, participants and metrics differ. Source definitions and evaluation context are provided in Supplementary Note S3 and Table S3.

Model selection depends on the mapping required between sensor signals and physical outputs, including temporal history, sparse spatial sampling and wearer-specific calibration. For history-dependent signals, recurrent or temporal-convolutional models can incorporate previous samples; tactile sequences have also been used to model hand–object dynamics [198]. Using 23 physical tactile sensing elements (taxels), a self-attention super-resolution model inferred 2700 virtual taxels and reported a localization root mean square error (RMSE) of 0.73 mm in robotic tests [199]. A digital-channel system localized contacts across up to 368 regions and used finite-element-informed neural decoding to reconstruct distributed forces, with a mean force-magnitude error of approximately 0.11 N from a 598-sample dataset (45 kB total) [200]. For human hand-pose reconstruction, a stretchable liquid-metal glove instead used hand-size-specific linear estimation with Kalman filtering. Its four hand-size/calibration conditions each involved a different participant [201] (Figure 6b).

Evaluation protocol changes the claim supported by a performance value. With the same dataset and convolutional-network configuration, ten-fold cross-validation yielded 99.85% accuracy, versus 85.1% under leave-one-subject-out testing [9] (Figure 6a). The observed 14.75-percentage-point protocol gap is not a causal estimate of leakage. Calibration creates another distinction: individualized, adaptive and generalized training of the lower-limb long short-term memory model gave root mean square errors of 2.81 ± 0.75°, 5.52 ± 2.10° and >10°, respectively [22] (Figure 6c). These summarize joint-angle outputs in a common single-participant test segment; the standard deviations are across joint-angle outputs, not participants. The >10° value remains a strict lower bound. Transformer estimators tested on unseen participants reported study-specific errors of 5–6° [202]. Wearable activity-recognition results depend on the validation method, which should therefore be explicit [203]. Differences in tasks, participants, metrics and validation conditions therefore prevent cross-study magnitudes from ranking intrinsic model capability.

### 7.3. Multimodal Sensor Fusion and Personalization

The strongest reason to fuse modalities is not data volume but failure-mode complementarity. IMUs provide inertial motion signals, but orientation estimates depend on drift control and sensor mounting. Textile strain and pressure channels capture local deformation but depend on fit. sEMG adds neuromuscular information unavailable from mechanical sensors, yet it is sensitive to motion artifact, perspiration, and crosstalk. Insole pressure measures loading more directly but remains footwear-dependent. Fusion is most defensible when these error sources are partly independent. Even then, the gain may be small: adaptive weighting of inertial and insole signals improved the F1 score for freezing-of-gait detection by 0.029 over the better single modality and by 0.008 over simple concatenation under subject-wise cross-validation [205]. A model combining insole pressure, motion-capture signals and EMG reported 96.00% gait-phase accuracy versus 94.67% for its EMG-only comparator; however, the published fusion rule references ground-truth labels, leaving label-free inference and the incremental gain unresolved [206].

The usual early-, feature-, and model-level fusion taxonomy says little about failure tolerance. One sEMG/IMU study simulated sensor failure by zeroing all channels from a selected sensor in the test set while leaving training data unchanged [207]. This tests artificial input removal, not physical detachment, laundering or wet degradation. The methods in Table 5 require channel-loss testing matched to intended device failure modes (Section 9.4); intact-input performance alone does not establish robustness.

#### Generalization, Personalization, and Calibration Burdens

Generalization should be reported along separate axes. An unseen user, a new session, re-donning, a different garment instance, an unseen task, an unseen object or contact location, a new environment, and transfer from healthy participants to a clinical population are not equivalent tests. Authors should state which axes are held out and which factors remain shared between training and test data.

Calibration is a deployment cost. Reports should state the minutes of calibration data, labelled samples per user, reference equipment required to generate those labels, whether the device was removed and re-donned between calibration and test, and the number of sessions involved. Missing quantities should be labelled “not reported” rather than reconstructed from indirect information.

Personalization can recover accuracy, but it shifts work to the new user. Fine-tuning often requires labelled data and a reference system that may not be available in routine use. Target-user adaptation and population-level pretraining address different data requirements. Contrastive self-supervised pretraining on 2700 s of unlabelled spontaneous motion, followed by five labelled repetitions per gesture class for each target user, yielded 91.7–94.2% directional-gesture accuracy from a thumb sleeve with two stretchable sensors [208]. Few-shot domain adaptation reached 97.59% cross-user gesture accuracy from five calibration samples per gesture and remained effective with one sample per gesture [209]. At population scale, self-supervised pretraining on 700,000 person-days of accelerometry yielded a median relative F1 improvement of 24.4% across eight benchmark datasets [210]. Personalized semi-supervised federated learning offers a related route for retaining user-specific models with fewer labels [211].

Training-population scale and user-specific adaptation shape generalization and calibration burden in different ways. In the 193-participant emg2pose benchmark, regression was evaluated on held-out users, held-out movement stages and their combination (Figure 6d); these were distinct test sets, not paired deterioration measurements, and estimation improved with more training participants [204]. A large-cohort wrist-sEMG interface supported closed-loop control with generic decoders in new users without participant-specific decoder training; wrist cursor control nevertheless required per-user normalization of the predicted velocity range [212]. For offline handwriting, adding 20 min of target-user data to a model pretrained on 6400 participants reduced character error rate by a reported median relative 16% [212]. In a separate 6527-participant model, personalization generally improved the target user’s performance but degraded transfer when applied to another user [212]. Few-shot pathological-gait modelling addressed a different population shift, using simulated pathological gait from healthy volunteers for pretraining and limited labelled data from four patients for adaptation [163]. These advances strengthen decoding across users and tasks, but their value in repeated wearable use depends on how changes in sensor placement, electrode–skin coupling and device instances affect task-level error and recalibration burden.

### 7.4. Real-Time Feedback and Human–Machine Interfaces

Once a system acts on its own prediction, mean offline error is no longer enough. Closed-loop operation adds end-to-end latency, confidence calibration, failure detection, and the consequences of an incorrect action. Delayed or overconfident feedback can degrade user interaction and become a safety concern when the output drives therapy or assistance.

Wearable systems now place sensing and feedback in the same physical interface. Self-powered electrotactile gloves deliver stimulation through the sensing garment [213]. Digitally embroidered gloves combine tactile sensing, vibrotactile actuation, and a user-specific haptic perception model, increasing tactile-occlusion accuracy from 5.7% to 88.6% [214]. Skin-integrated electrotactile interfaces first map each user’s sensation threshold, then encode contact against that map [215]. Personalization also extends to control: a user-specific intent detector driving a soft shoulder exosuit increased shoulder range of motion by 17.5° and reduced trunk compensation in participants after stroke or with amyotrophic lateral sclerosis [216]. Co-locating sensing and actuation can introduce electrical and mechanical crosstalk, so the problem remains partly materials- and device-level even when the controller is learned.

**Figure 7:**
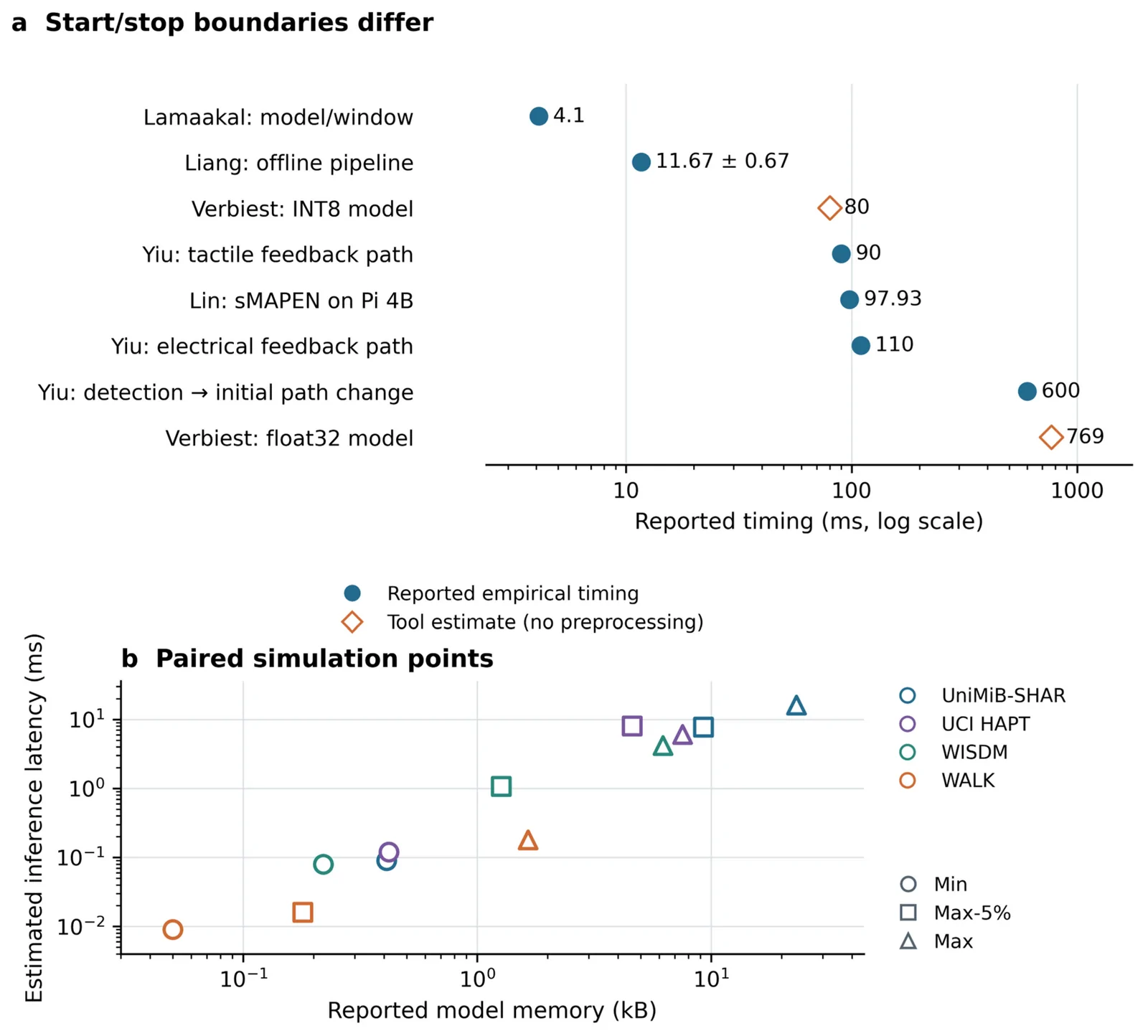
Timing boundaries and paired simulation-derived inference costs. (**a**) Source-reported empirical timings (filled circles) and Edge Impulse tool estimates excluding preprocessing (open diamonds) are shown on a logarithmic axis. The estimates are 769 and 80 ms for 32-bit floating-point (float32) and 8-bit integer (INT8) models, respectively, on the nRF52840 target [219]. Tactile (90 ms) and electrical (110 ms) feedback-path responses and the 600 ms interval from obstacle detection to initial flight-path change have different timing boundaries [218]. The offline pipeline value is 11.67 ± 0.67 ms [220]; the ± statistic, timing hardware and complete acquisition delay are not established. Model timings are 97.93 ms for the multi-scale attention patching encoder network (sMAPEN) on Raspberry Pi 4B [222] and 4.1 ms per window for INT8 TinyKAN-HAR (Kolmogorov–Arnold network for human activity recognition) on an 80 MHz Cortex-M4F-class microcontroller [223]; neither is acquisition-to-action latency. (**b**) Twelve discrete paired memory/latency configurations from four datasets [221] are plotted individually. Latencies derive from simulated cycles under Quentin assumptions (205.1 MHz, 0.54 V), not measured deployment. Dataset colours and configuration symbols identify the reported pairings. Because tasks, hardware, timing boundaries and provenance differ, the plotted values are not ranked across studies and no universal response-time threshold is asserted. Source definitions and resource quantities are provided in Supplementary Note S4 and Table S4; the twelve exact paired configurations are provided in Supplementary Note S5 and Table S5.

Latency figures are interpretable only when the measured interval is defined. The 40 ms value in [217] is described as both end-to-end and processing/classification time, leaving its timing boundary ambiguous. In a drone interface, tactile and electrical feedback-path responses were 90 and 110 ms, whereas the 0.6 s interval ended at an initial flight-path change rather than completed correction [218]. Tool-based nRF52840 (Cortex-M4F, 64 MHz) inference estimates excluded preprocessing, while static-buffer tests omitted live inertial acquisition [219]. The 11.67 ± 0.67 ms offline knee-angle result left hardware and the variability statistic unspecified [220]. Figure 7 contrasts twelve simulated memory–latency configurations [221] with model-only inference timings [222,223]; model-file size, deployed flash and random-access memory (RAM) describe different resources [224]. These differences preclude ranking end-to-end responsiveness or establishing a universal 100 ms deadline.

Decision coverage is a separate requirement for closed-loop suitability. An evidential sEMG grasp classifier distinguished insufficient (vacuity) from conflicting (dissonance) evidence: under dual-threshold rejection, a true-acceptance rate of 83.51% among retained predictions came with 81.43% of inputs rejected (eight participants, ten runs); no-rejection accuracy was 51.44% [225]. Conformal prediction instead reported calibrated intervals for continuous core-body-temperature estimates under heat stress [226]. Assessing closed-loop suitability therefore requires both a defined action-delay boundary and decision coverage under uncertainty.

### 7.5. Section Synthesis: Generalization, Robustness, and Closed-Loop Requirements

Sensor physics should guide both algorithm design and the tests used to evaluate it. Hysteretic and viscoelastic responses depend on deformation history, providing a rationale for temporal models, while unreliable channels motivate masking or weighting. Multimodal fusion offers a complementary approach when different inputs compensate for one another, but its practical value depends on preserving the intended estimate as signal quality degrades, rather than simply improving accuracy with all channels intact.

This relationship between signal quality and inference also shapes what generalization means. Subject-independent evaluation tests transfer to unseen users, but re-donning and session drift can alter body coupling and calibration even for the same person. Changes in tasks, devices or environments, including the loss of a sensing modality, may further affect the learned relationship between signals and outputs. The relevant question is therefore whether the model maintains reliable estimates under the conditions of use and how much recalibration this requires. When those estimates guide feedback, their reliability must also extend to the timing and handling of uncertain or failed inputs, ultimately supporting the intended functional response. Section 8 examines which application-level claims this evidence can justify.

## 8. Application-Level Claim–Evidence Synthesis and Translational Gaps

### 8.1. Application-Level Claim–Evidence Framework

Section 3, Section 4, Section 5, Section 6 and Section 7 evaluated layer-specific mechanisms and failures. Section 8 asks what the integrated system can legitimately claim, building on the device and review scope established in [1,227,228,229,230,231,232,233]. Interpretation depends on the claimed output, population, reference, setting and duration. Table 6 is the main synthesis: it separates the application claim, the validation context actually demonstrated and the evidence still required for a stronger claim.

### 8.2. Physiological and Biochemical Monitoring

Physiological and biochemical monitoring differ in how recorded signals relate to the claimed biological quantity. In EDA and biopotential recording, electrode geometry, skin contact and motion can affect signal quality [91,92,93,94,95,96,98,234,235], whereas deformation, temperature and humidity changes are intended inputs in respiratory or multifunctional wearables [167,169,236,237,238]. These distinctions limit what can be inferred from improved materials or signal processing alone. Biofluid sensing additionally depends on sampling and fluid composition, making selectivity, fouling and calibration stability central [2,239,240,241]. Screen-printed textile glucose-sensing [109] and batteryless wireless glucose monitoring [242] illustrate integration approaches, whereas body-matched electromagnetic glucose monitoring [243] uses a different, indirect sensing principle. Application claims therefore require a suitable measurement reference for the claimed output, population and conditions of use (Table 6).

### 8.3. Optical and Multimodal Healthcare Sensing

Flexible photodetectors, body-worn optical demonstrations and physiological measurement literature provide different forms of evidence along the path from optical detection to an interpretable output [244,245,246,247,248]. Tissue scattering, placement and illumination can alter body-worn signals, so detector sensitivity is a valid device-level result, whereas a physiological measurement claim requires agreement with an appropriate reference under those conditions. Multimodal sensing raises a related attribution problem: when an additional channel is claimed to improve the target output, its contribution must be distinguished from the performance of the existing inputs. A comparable single-modality baseline, assessed against the same output reference and timing conditions, can test that contribution; robustness claims additionally require evaluation as inputs degrade. The application-level question is what validated output the integrated device supports in the intended setting.

### 8.4. Human Motion, Human–Machine Interaction, and Assistive Applications

Measurement or estimation, intent/interaction inference, feedback and functional benefit are distinct claims. Textile gloves and knee sleeves illustrate movement estimation [165,174]; tactile interfaces and sensing-feedback demonstrations address interaction [185,249,250,251,252,253,254,255]. Gesture or classifier performance supports the tested classification or interaction task; assistive or rehabilitation benefit requires functional outcomes in intended users. Laboratory feasibility in healthy participants provides a different evidence context from intended-user effectiveness [256,257,258,259]. Few-shot pathological-gait modelling [163] concerns prediction, while interviews after stroke [260] identify user preferences rather than demonstrate recovery. For human-facing feedback demonstrations, evaluation should address task-relevant timing and applicable safety and acceptance concerns; claims of functional or clinical benefit additionally require meaningful outcomes in the intended population. For repeated-use claims, calibration burden and generalization should be assessed across relevant users, sessions and re-donning conditions. Table 6 separates engineering and intended-user evidence so that stronger functional claims are matched to the relevant outcomes.

**Table 6:** Application-level claims, validation contexts, and remaining evidence requirements across wearable sensing. Examples include direct, transferable, and system-comparator evidence as defined in Section 2.3.

Application Domain	Representative Application-Level Claim	Evidence Context and Representative Studies	Remaining Translational Evidence Needs
Physiological monitoring	Continuous or repeated body-worn measurement of electrophysiological and vital-sign signals, including ECG, EEG, EMG, pulse/PPG, respiration, and temperature.	Selected soft and nanomaterial-enabled interfaces have undergone controlled on-body human validation, including comparison with conventional or clinical reference measurements [150,234,235]. Evidence ranges from short wear demonstrations to controlled participant studies.	Motion-artifact robustness; stable skin coupling and drift control; repeated-use reliability; intended-population validation; longer-duration reference-based evaluation.
Biochemical monitoring	Biofluid-based measurement of metabolites, nutrients, electrolytes, pH and related biomarkers, alongside indirect body-coupled glucose estimation.	Biochemical sensing examples include integrated sweat measurements [2,240], a flexible battery-free glucose-sensing system [242], and body-matched electromagnetic glucose monitoring [243]. Validation contexts and sensing mechanisms differ.	For biofluid assays: sampling variability, evaporation, fouling, selectivity and sensor/enzyme stability where relevant; across approaches: calibration, reproducibility and reference validation matched to the claimed output.
Optical sensing	Flexible optical measurement of biosignals, photodetection, activity, fluorescence, or spectroscopy-related outputs.	Evidence spans body-worn biosignal demonstrations and device-level flexible photodetector studies [244,245,247,248]; these studies do not provide equivalent human or clinical validation.	Ambient-light and tissue-scattering robustness; positioning and optical coupling; power and encapsulation; long-term stability; direct human-reference validation for the intended claim.
Human–machine interaction, haptics, and assistive systems	Inference of gesture, hand motion, intent, or tactile interaction, with optional bidirectional haptic or assistive feedback.	Evidence includes wearable engineering demonstrations, multimodal sensing-feedback systems, and selected intended-user testing, including smart-textile and prosthetic interfaces [10,165,249,250,252].	Cross-user generalization and calibration; re-donning robustness; latency and channel or modality loss; feedback safety and acceptability; evidence of meaningful functional benefit where such benefit is claimed.
Biomechanical and functional monitoring	Measurement or estimation of gait, joint motion, loading, muscle activity, movement quality, and functional activity.	Evidence includes controlled reference validation, intended-user cohorts, and separate free-living feasibility assessments [159,161,162]. Some systems serve as comparators rather than direct nanomaterial-enabled evidence.	Appropriate concurrent ground truth; repeated-session reliability; calibration retention; technical-failure reporting; diverse intended-user cohorts; clear separation of real-world feasibility from real-world accuracy.
Digital-health deployment	Longitudinal monitoring, digital biomarkers, adherence measures, remote rehabilitation, or decision-support outputs from connected wearable systems.	Evidence includes home-based rehabilitation, free-living gait assessment, large-scale wearable datasets, and cloud-connected systems [156,161,179,210,252], with substantial reliance on transferable or system-comparator evidence.	Interoperability and privacy/security; calibration retention; charging/connectivity burden; energy availability and autonomy; missing data and adherence; explainability and workflow integration; sustained data quality and longitudinal clinical relevance.

Abbreviations: ECG, electrocardiography; EEG, electroencephalography; EMG, electromyography; PPG, photoplethysmography.

### 8.5. Digital-Health Deployment and Longitudinal Evidence

Longitudinal monitoring changes the question from whether a device performs in one session to whether its measurements remain interpretable across repeated-use. Home and free-living wearable studies [156,161,261], alongside related home-rehabilitation and review evidence [179,256,257,258,259], inform feasibility within their respective contexts, while accuracy claims require suitable concurrent references. Re-donning may alter calibration, and non-wear or acquisition failures can change which observations enter a longitudinal trend. Model-derived estimates must therefore retain their reference and timing boundaries [217,218,219,225,226], while clinical interpretation also depends on the relevant healthcare context [262,263]. Adherence and workflow compatibility, including privacy and failure handling, shape whether such longitudinal evidence can be collected and used.

Power availability affects the same continuity of evidence. Flexible harvesting [264,265,266] and storage strategies [53,267,268,269] contribute when they sustain the sensing, communication and computing workload under representative illumination, deformation and wear. Peak component output alone cannot establish the availability of interpretable data over time.

### 8.6. Cross-Application Evidence Gaps

Across applications, what a wearable demonstrates depends on the meaning assigned to its output. Reference agreement can support a measurement claim, but intended-user benefit requires functionally relevant outcomes. Under repeated-use, changing body coupling or data availability can further alter the meaning of apparent trends. The resulting evidence needs are complementary rather than sequential, consistent with the overlapping E1–E5 contexts. Section 9 sets out the reporting conditions needed to interpret these claims, while Section 10 proposes nonlinear research priorities and evidence targets for addressing the remaining translational gaps.

## 9. Proposed Cross-Layer Reporting Framework for Claim–Evidence Alignment

Across Section 3, Section 4, Section 5, Section 6, Section 7 and Section 8, reported metrics capture different parts of the sensing chain and therefore support different kinds of claims. Material sensitivity, for example, characterizes a transducer under specified conditions, whereas a body-worn estimate must be judged against its reference and acquisition context. Combining those results into a universal score would obscure what each study actually demonstrated. Table 7 instead identifies the information needed to interpret performance in relation to the claim being made.

### 9.1. Material, Device, and Biointerface Performance Metrics

Sensitivity or conductivity without device geometry and relevant environmental, mechanical and biological conditions cannot support a claim of stable sensing. Range, hysteresis, drift and durability determine whether a peak response remains usable; reproducibility, safety and manufacturing compatibility constrain translation [19,29,47,70,71]. Table 7 lists the corresponding fields.

Biointerface performance belongs to the complete electrode stack, not the isolated material. For electrophysiological interfaces, relevant tests include frequency-dependent impedance magnitude and phase, noise or SNR, drift and motion artifacts; electrochemical measurements require analyte- and readout-specific assessment (Section 8.2). Independent dry-electrode experiments link contact compliance and adhesion to changes in interface impedance and motion artifacts [234]. Flexible and textile EDA and ECG studies separately demonstrate geometry- and skin-contact-dependent signal quality [91,92,93,94,95,96], while ambulatory EEG documents movement-related artifacts [98] and EEG phantom tests isolate cable-sway and electrode-area effects [270].

Simulation, benchtop or phantom tests, acute or chronic in vivo studies, and human or longitudinal clinical evaluations support different claims. Acute interface performance does not establish chronic biological stability, and human feasibility does not establish intended-population validity [5,8,99,112,121].

### 9.2. System-Level Wearable, Wireless, and Power Metrics

Complete-system claims depend on the path from transducer to usable output, including body coupling, readout, power, calibration and failure handling. Placement, fit, task and test conditions must remain linked to the participant population and whether the reference was simultaneous [6,8,121,156,161]. Simulated network optimization couples relay placement, transmission power and time allocation [139], whereas deployment reports must independently document link reliability under body motion.

Wireless range is interpretable only when measured and calculated values are distinguished and the medium, reader orientation and body or deformation conditions are stated. Passive-array scaling also depends on channel count, spacing and decoding of overlapping resonances. Readout and telemetry claims must account for conditioning, multiplexing, encapsulation and crosstalk among sensing or stimulation paths [6,127,130].

Peak harvesting output does not establish autonomy: sustained power must reach the actual sensing or communication load at the stated duty cycle and duration [7,142,143,144,145,146,147,269]. For photovoltaic (PV) systems, illumination spectrum or intensity, collection area, harvested and stored energy, and power-management efficiency are needed to interpret the effects of orientation, shading and indoor/outdoor exposure [264,267,268]. Table 7 retains the complete power-reporting inventory.

### 9.3. Human-Participant, Clinical, and Deployment Validation

Human and deployment claims depend on the population, reference, setting and duration tested. A multisensor gait system showed agreement with optical tracking in structured laboratory tests but was evaluated for usability, not independent-reference accuracy, during 2.5 h of unsupervised use [161]. Stroke-rehabilitation trials [177,178,179] and a clinician-rated Parkinson’s assessment glove [180] examined different human endpoints.

References must match the claim: angle, orientation or trajectory for kinematics; force or pressure for loading; and functionally or clinically meaningful outcomes for claims of functional or clinical benefit. Participant number, intended population, task, reference method, duration and relevant repeated-session reliability constrain the inference. Benchtop, acute, chronic and human evidence cannot be substituted for one another [8,160,161,162,177,178,179,180].

Deployment readiness includes sustained operation and acceptable user burden, rather than accuracy alone [156,161,179]. Table 7 identifies the calibration, missing-data, comfort, adherence, safety and maintenance fields. Feedback and workflow requirements must come from intended users, as stroke-rehabilitation interviews illustrate [260,271].

### 9.4. Algorithmic Generalization, Multimodal Robustness, and Closed-Loop Validation

Participant overlap and split definition determine what generalization a score supports. Subject-mixed, leave-one-subject-out and external-cohort tests answer different questions. Subject-dependent and independent results require sample size and overlap information; a gap between otherwise identical models may reflect the partitioning protocol [9,22,204,212].

Robustness reporting should specify the sensor failure mode and held-out axis (Section 7). Sparse tactile reconstruction [199], transfer between replaceable robotic-skin instances [193] and IMU–insole fusion gains over a unimodal baseline [205] establish different capabilities. To substantiate graceful degradation, studies should test matched intact and degraded conditions, distinguishing physical sensor changes from synthetic channel removal and reporting task-level error, recalibration burden and, where predictions can be withheld, decision coverage.

Real-time and closed-loop claims require instrumented timing. Reports should separate inference-only, feedback-path, and complete sensing–inference–actuation latency, identify the hardware and compute location, and state calibration burden. When predictions drive an action, confidence calibration, uncertainty, abstention, and failure detection become part of the control specification [217,218,219,225,226].

**Table 7:** Cross-layer reporting framework for claim–evidence alignment in functional nanomaterial-enabled wearable and bioelectronic systems. The entries synthesize Section 3, Section 4, Section 5, Section 6, Section 7 and Section 8 and are intended to support consistent interpretation, not to define a formal reporting standard.

Assessment Domain	Key Reporting Elements	Interpretive Purpose	Evidence Insufficient for Stronger Claims
Material and device performance	Composition; fabrication method; substrate; transduction mechanism; device geometry; sensing range; hysteresis; drift; cyclic durability; environmental stability.	Places material-level performance in the context of the integrated device and the conditions under which it was tested.	Peak sensitivity or conductivity reported without sensing range, durability, environmental stability, or device-integration context.
Biointerface performance	Active material; electrode area/thickness; substrate and encapsulation; modality-appropriate impedance magnitude/phase or electrochemical response; noise or SNR; drift; bending stiffness; adhesion; strain tolerance; motion artifact; cyclic fatigue.	Links electrical or electrochemical performance to the mechanical and biological quality of the complete interface.	Isolated-material or single-frequency impedance results used to infer interface performance without tests relevant to the intended skin- or tissue-coupled measurement.
Reproducibility and manufacturability	Batch-to-batch reproducibility; process control; scalable fabrication; cost; material safety; sterilization compatibility where relevant.	Connects laboratory performance with the ability to reproduce and scale the device for sustained or clinical use.	A scale-up or translational claim without process reproducibility, manufacturing consistency, safety, or sterilization evidence where relevant.
Validation context	Simulation/calculation; benchtop; proxy/phantom; acute in vivo; chronic in vivo; on-body human; intended-user/clinical; longitudinal or real-world evaluation.	Aligns claim strength with the type and context of validation actually demonstrated.	Chronic-use claims based only on acute tests, or human clinical or deployment claims extrapolated from simulation, benchtop, proxy or preclinical evidence without claim-matched human evaluation.
Wireless and RF performance	Measured versus calculated range; test medium; reader/antenna orientation; body or deformation condition; channel count/spacing; direct versus model-based decoding.	Determines whether wireless or passive readout remains usable under realistic body-coupled and scaled conditions.	A range or passive-array claim without the test medium, orientation, deformation/body condition, channel configuration, or decoding method.
Power and energy autonomy	Peak versus sustained average power; actual load; duty cycle; storage strategy; supported sensing/communication function; operating duration; for solar/PV, illumination conditions, active area, harvested/stored energy, and power-management efficiency.	Determines whether the energy source sustains the integrated wearable function rather than only generating component-level output.	A self-powered or autonomous-system claim based only on peak harvesting output without sustained power delivered to the actual load or, for PV systems, without representative illumination conditions.
Integrated wearable and human validation	Body placement and fit; calibration; participant population; task; simultaneous reference; error/agreement metrics; repeated-session or re-donning reliability; technical failures.	Separates complete wearable-system performance from component characterization and short-term feasibility in healthy participants.	Claims of intended-user validity or sustained use without the relevant population, reference, calibration, repeatability, or technical-failure evidence.
Algorithmic generalization and closed-loop operation	Validation split and overlap across participants, sessions and device instances; subject-dependent versus subject-independent performance; calibration and re-donning burden; channel/modality-loss testing; intermodal synchronization where applicable; latency scope; hardware and compute location; uncertainty calibration or abstention.	Determines whether model performance generalizes beyond the development dataset and whether closed-loop operation is technically and functionally credible.	Cross-user generalization inferred from participant-mixed splits; repeated-use robustness inferred from single-session testing; failure tolerance inferred from complete-input tests; or real-time operation claimed without instrumented, task-relevant timing.
Deployment readiness	Home or long-term duration; calibration retention; charging/connectivity burden; usable-data coverage and reasons for missingness; comfort; adherence; safety; maintenance; user/clinician interpretability; workflow fit.	Assesses whether performance can be sustained outside controlled laboratory conditions with acceptable user and system burden.	A deployment-readiness claim based on short-term feasibility without evidence on calibration retention, usable-data coverage, missingness, burden, adherence, safety, maintenance, or workflow integration.

Abbreviations: PV, photovoltaic; RF, radio frequency; SNR, signal-to-noise ratio.

## 10. Challenges, Open Questions, and Translational Roadmap

Performance demonstrated at the material- or device-level may change when the technology is integrated into a body-coupled system. Changes in coupling or signal acquisition can propagate through subsequent layers, so a limitation revealed during use may require revisiting earlier design choices. Section 10 translates these dependencies into coupled experiments that test performance under intended-use conditions. Table 8 presents the corresponding evidence targets as revisitable engineering milestones rather than a linear readiness scale.

### 10.1. Material Reliability, Biointerface Stability, and Scalable Fabrication

Test material and biointerface reliability under use-relevant mechanical, environmental and biological exposure. For wearables, select deformation, sweat, washing, abrasion, temperature or humidity cycling, and adhesion tests according to intended use [19,47,70,71,101]. For implants, assess tissue micromotion, foreign-body responses, electrochemical degradation, and encapsulation or insulation lifetime [5,87,88,99,112].

Co-design fabrication and interface performance, then test process control and batch reproducibility. Safety, packaging, assembly, calibration, maintenance and sterilization where required determine whether performance is reproducible outside the originating laboratory [3,19,47,100,102,103]. The fabrication routes and device examples in Section 3 and Section 4 establish the mechanisms; the next priority is reproducibility under the intended use.

### 10.2. Integrated Wearable and Bioelectronic Systems, Wireless Readout, and Power

Test readout, power, telemetry and packaging together with the sensing interface. Experiments under movement and body loading should establish usable wireless range and passive-channel separability while delivering sustained power to the actual sensing, processing and communication load at realistic duty cycles [6,7,121,127,130,142,143,144,145,146,147]. Joint network and energy optimization remains a system-level problem [139,272].

Evaluate PV or hybrid harvesting against the platform’s actual power demand under representative light availability, orientation and shading. Solar-storage and multi-harvester combinations may reduce intermittency, but autonomy requires adequate storage, regulation and energy delivered to the load [264,267,268]. Flexible conductors and solar-cell devices address the component layer [74,266]; Section 9 specifies the illumination, area, energy, efficiency and duty-cycle reporting needed for complete-system tests.

Test multimodal bioelectronics for stimulation artifacts, photoelectric coupling, thermal loading and cross-channel interference, including EMG–cortisol crosstalk [105]. Chronic implant and gut–brain systems require longer-term tests of insulation, packaging, wireless power/telemetry and biological endpoints beyond acute proof-of-concept [55,75,116,119,120].

### 10.3. Human Validation, AI-Enabled Interpretation, and Closed-Loop Deployment

Prioritize longitudinal studies in intended populations, with concurrent references for real-world measurement claims and functional outcomes for rehabilitation claims. For gait sensing, extend structured reference comparisons to representative free-living tasks and repeated re-donning [161]. For home rehabilitation, report adherence, treatment dose and functional change against suitable comparators [177,178,179]. Stroke-rehabilitation interviews inform feedback and workflow design priorities [260,271].

Compare calibration-free inference and few-shot adaptation against instrumented references under channel or modality loss, especially for continuous cross-user kinematic and force estimation [9,191,193,197,199,201,204,205,208,209,210,212]. Use matched intact and degraded inputs, state the permitted calibration data, and keep adaptation data separate from final evaluation data for the held-out users, sessions or device instances (Section 7.3). Report task-level error and recalibration burden (Section 9.4).

Evaluate closed-loop systems for meaningful behavioural, functional or clinical benefit, with uncertainty or abstention and failure handling. Instrumented end-to-end timing and compute location must support the intended action (Section 7 and Section 9). Studies should also evaluate user/clinician burden, workflow fit, privacy and safety. Where feasible, matched tests with and without feedback under realistic sensing and link conditions can assess its additional functional value.

### 10.4. Cross-Layer Translational Roadmap

Table 8 links observed limitations to targeted experiments and design decisions. If a passive resonant array loses channel separability during wear, tests of body-dependent reader coupling and resonance overlap can help distinguish readout limitations from inference errors and direct redesign toward the limiting subsystem. Representative patent publications [273,274,275,276,277,278,279,280,281,282], summarized in Supplementary Table S7, provide complementary examples of potential integration and manufacturing pathways; their suitability for intended use still requires appropriate testing.

### 10.5. Limitations of the Review

This review is a structured cross-layer critical synthesis rather than an exhaustive systematic or scoping review. Searches were adapted to the terminology of different domains and focused primarily on English-language records within the documented coverage windows, with targeted supplementary and foundational sources used for cross-cutting questions. Additional searches for MXenes, graphene, metal–organic frameworks and hydrogels increased the visibility of these classes relative to materials not searched in the same way. Retrieval counts, citation frequency and the prominence of particular topics therefore cannot establish field prevalence or comparative maturity.

Even within the selected literature, differences in endpoints, reference methods and operating conditions limit comparison across the sensing chain. Transferable studies and system comparators can clarify design and validation problems without providing direct evidence for nanomaterial-enabled wearable performance, while heterogeneous outcomes preclude pooled quantitative estimates. The proposed frameworks and roadmap therefore help align claims with their supporting evidence and identify the tests still needed, rather than defining consensus standards or exhaustive rankings. Existing standards and guidance address related questions but do not define the overlapping E1–E5 evidence contexts (Section 6.5).

## 11. Conclusions

Functional nanomaterials have expanded the signals accessible to wearable and bioelectronic devices, but component performance depends on the conditions under which it is measured. Hysteresis, drift and aging constrain the useful material-service window; fabrication, encapsulation and contact with skin or tissue affect the signal entering readout. Passive RF arrays introduce reader–sensor coupling and channel-separation constraints, while active electronics shift demands to power, communication and computation. These effects can propagate into missing or distorted inputs before inference begins. An inaccurate application-level estimate may therefore originate upstream, even when the inference model performs well on intact laboratory signals.

Research priorities should begin with the intended claim: the measurand, target population, reference, operating conditions and service window determine which failure modes require testing. Re-donning can alter textile–body coupling, whereas long-term implants require evidence of biological and encapsulation stability. Integrated evaluations should test measurement consistency across independent device instances and repeated-use, assess fabrication reproducibility, and report calibration requirements and user burden. Power delivery, communication, computation and, where relevant, feedback should be co-designed for sustained workloads and evaluated under realistic link, exposure, resource and latency constraints. When performance deteriorates, targeted experiments should identify the limiting subsystem and guide redesign.

Population-scale training and user-specific adaptation have improved decoding under defined evaluation protocols. Repeated-use validation must additionally test how sensor placement, electrode–skin coupling, device instances and channel availability affect task-level error and recalibration burden. When predictions guide actions, acquisition-to-action delay and retained decision coverage under uncertainty must be evaluated alongside power and communication constraints. Free-living feasibility, reference-based measurement agreement, and functional or clinical benefit remain distinct achievements, requiring evidence matched to the claimed population, setting, outcome and duration. The complementary E1–E5 contexts and the reporting and experimental priorities proposed here organize this evidence without imposing a linear readiness scale. An integrated system thus supports its intended claim through evidence connecting the sensing pathway to the measurement, decision or functional outcome delivered during use.

## Figures and Tables

**Table 1:** Positioning of the present review relative to recent reviews spanning functional materials, wearable and bioelectronic systems, wireless integration, artificial intelligence, and clinical translation. The comparison summarizes stated scope and substantive coverage and is intended for positioning rather than quality ranking.

Review	Scope and Covered Layers	Positioning Relative to This Review
Shajari et al. (2023) [11]	AI-enabled wearable sensors for digital health, including wearable sensing materials and architectures, machine-learning interpretation, and healthcare applications.	Connects wearable sensing with AI and digital health; the present review instead organizes the evidence around cross-layer information fidelity, failure propagation, and claim–evidence alignment.
Kong et al. (2024) [12]	Wireless technologies for flexible and wearable sensing, spanning functional materials, wireless modules, system integration, data transmission, and applications.	Centres on wireless technologies and system integration; the present review treats wireless readout and power as one layer within a broader material-to-validation pathway.
Hong et al. (2025) [13]	Biomaterials for reliable wearable health monitoring, emphasizing hydrogels, fibres, hybrid biomaterials, durability, and tissue-facing interfaces.	Emphasizes material and body-interface reliability; the present review extends this perspective downstream through readout, interpretation, validation, and deployment.
Ren et al. (2025) [14]	Emerging-nanomaterial wearable bioelectronics for telehealth, including soft body integration, wireless functions, and remote health applications.	Provides a nanomaterial-to-telehealth systems perspective; the present review additionally separates failure modes and evidence requirements across intermediate system layers.
Mu et al. (2025) [15]	Wearable sensor design and materials integrated with machine learning for human–machine interaction.	Focuses on sensor and machine-learning approaches for human–machine interaction; the present review links algorithmic interpretation to physical sensor failure modes, validation strategy, and downstream claims.
Cheng et al. (2025) [16]	Flexible wearable sensors for real-time health monitoring, spanning materials, devices, system integration, and healthcare applications.	Provides broad materials-to-system coverage; the present review makes cross-layer information preservation and claim-matched validation explicit organizing principles.
Cherian et al. (2026) [17]	Skin-interfaced wearable sensing for clinical physiology and remote monitoring, including device validation and barriers to clinical deployment.	Emphasizes clinical translation and validation; the present review integrates this logic with material, biointerface, wireless/power, platform, and AI layers.
Suryaprabha et al. (2026) [18]	Wearable and implantable biosensors for continuous health monitoring, including flexible materials, biocompatibility, body integration, multimodal sensing, AI, and translation challenges.	Provides broad materials-biointerface-AI coverage; the present review explicitly tracks information degradation, failure propagation, and evidence strength across sequential system layers.
Present review	Functional nanomaterials, biointerfaces, wireless readout and power, integrated wearable platforms, AI-enabled interpretation, application-level claims, and human-centred validation.	Cross-layer synthesis framework connecting material-to-application information fidelity, failure propagation, and claim–evidence alignment.

**Table 4:** Wearable-platform categories, validation contexts, and translational limitations discussed in Section 6. The cited studies are representative examples and do not form a one-row-per-study inventory.

Platform Category	Representative Studies and Systems	Validation Context	Key Translational Limitations
Accessory and inertial systems	Smartwatches, full-body inertial systems, and compact IMU-based gait platforms [8,154,155,156].	Controlled comparisons with optical motion capture or electronic walkways, with selected home-based and clinical applications.	Placement and calibration dependence; limited concurrent reference measurements outside the laboratory; intended-user validation varies across studies.
Foot-worn platforms	Pressure/inertial insoles, textile socks, and smart-shoe systems [157,158,159,160].	Evidence spans healthy or simulated gait tasks, repeatability testing, and evaluation in Parkinson’s disease.	Sensitivity to footwear, walking surface, speed, sensing coverage, and drift; intended-user and longitudinal evidence remain uneven.
Textile garments	Optical-fibre smart pants and seamlessly knitted multisensor garments [164,166].	Proof-of-concept human studies with laboratory reference measurements, generally involving one or very few participants.	Limited evidence on fit, fabric mechanics, re-donning, calibration transfer, washability, and cross-user generalization.
Joint sleeves and braces	Textile braces and sleeves with strain or nanocomposite sensing, dual-IMU/ToF assessment, or thermal/compression actuation [170,171,172,173].	Engineering and healthy-participant evaluation, including reference-based motion testing and selected durability assessments.	Limited intended-user, clinical, repeated-session, and longitudinal validation; several systems remain primarily at the integration or proof-of-concept stage.
Skin interfaces and electronic skins	Adhesive hydrogel and epidermal interfaces integrating strain and sEMG sensing [175,176].	Small laboratory studies in healthy or incompletely characterized participant groups, with motion or functional reference measurements.	Adhesion, sweat exposure, biofouling, repeated attachment, cohort reporting, and lack of intended-population validation.
Smart gloves and hand-function systems	Clinical, rehabilitation, assessment, and engineering gloves integrating inertial, pressure, bending, or optical sensing [177,178,179,180,181,182].	Evidence ranges from engineering prototypes and laboratory reference testing to clinical assessment, randomized interventions, and home use.	Variable sample size, task specificity, calibration burden, participant reporting, and cross-user generalization; long-term functional benefit remains inconsistently established.
Haptic, tactile, and prosthetic systems	Prosthetic vibrotactile feedback, gait-triggered haptic stimulation, and multimodal sensing-feedback gloves [10,184,185].	Evidence includes intended-user prosthetic testing and preliminary healthy-participant or component-level demonstrations.	Limited longitudinal evidence for functional benefit, safety, acceptance, repeated-use reliability, and workflow integration in intended populations.
Multimodal gait and mobility systems	Platforms combining plantar-pressure, inertial, and distance sensing for gait and propulsion assessment [161,162].	Controlled laboratory validation in gait-disorder or post-stroke cohorts, with separate feasibility testing during habitual or out-of-laboratory activity.	Laboratory accuracy and free-living feasibility are not equivalent; synchronization, personalization, sensor failure, dataset reuse, and lack of concurrent real-world ground truth remain important limitations.

Abbreviations: IMU, inertial measurement unit; sEMG, surface electromyography; ToF, time of flight.

**Table 5:** Cross-layer mapping between soft-sensor failure modes, candidate algorithmic responses, and claim-matched validation. The mapping is an author synthesis of the reviewed evidence and is not prescriptive.

Failure Mode	Underlying Source	Candidate Algorithmic Strategy	Claim-Matched Validation
Hysteresis and viscoelastic lag	Percolation-network relaxation and matrix viscoelasticity in elastomeric and textile composites (Section 3.1 and Section 3.4).	History-aware sequence models, including LSTM, GRU, TCN, or Transformer architectures, rather than memoryless mappings.	Report errors separately during loading and unloading and evaluate performance across relevant strain rates.
Baseline drift	Creep, temperature/humidity sensitivity, sweat ingress, and fibre fatigue (Section 3.2 and Section 3.4).	Drift-aware normalization, recursive baseline updating, and self-supervised recalibration from unlabelled wear data.	Multi-session and multi-day evaluation with controlled wear conditions and explicit characterization of temporal drift.
Mounting and re-donning variability	Garment tension and sensor displacement relative to anatomy between wears (Section 6.1 and Section 6.3).	Domain adaptation, few-shot recalibration, and placement-invariant feature representations.	Remove and re-don the device between training/calibration and testing, preferably across repeated sessions.
Missing or saturated channels	Interconnect fatigue, wash damage, or out-of-range loading in dense sensor arrays (Section 3.4 and Section 6.3).	Attention-based channel weighting, masked or channel-dropout training, and graceful-degradation strategies.	Identify whether channel loss is simulated or experimentally observed, specify the affected channels, and compare task-level performance with a matched complete-input condition.
Spatially sparse sampling	Finite taxel or electrode density over gloves, sleeves, insoles, or other body-worn interfaces (Section 6.2, Section 6.3 and Section 6.4).	Hierarchical models, learned spatial priors, and cross-modal supervision.	Evaluate held-out contact locations and unseen objects rather than only unseen repetitions.
Inter-user variability	Differences in anatomy, soft-tissue mechanics, movement strategy, and pathology (Section 6.1 and Section 6.5).	Population-level modelling, transfer learning, domain adaptation, and rapid personalized fine-tuning.	Use subject-independent evaluation (e.g., leave-one-subject-out), preferably with an external cohort where feasible, and report the performance change relative to subject-dependent evaluation.

Abbreviations: GRU, gated recurrent unit; LSTM, long short-term memory; TCN, temporal-convolutional network.

**Table 8:** Cross-layer translational roadmap for trustworthy wearable sensing. Milestones are nonlinear and may be revisited; AI-based interpretation and closed-loop personalization apply only where the target application requires them.

Roadmap Milestone	Key Translational Challenge	Priority for Future Work	Evidence Target
1. Material and device reliability	Peak material performance does not ensure stable sensing after device integration, repeated deformation, or environmental exposure.	Establish reproducible fabrication and application-specific durability testing under relevant mechanical, thermal, humidity, sweat, washing, or other environmental conditions.	Stable, calibrated device response after integration and repeated exposure under conditions representative of the intended use.
2. Biointerface stability and device integration	Mechanical mismatch, adhesion, impedance, encapsulation, micromotion, and biological stresses can destabilize epidermal and implantable interfaces.	Co-design the active material, electrode geometry, substrate, encapsulation, mechanical compliance, sterilization strategy where relevant, and biological safety.	Integrated interface with documented electrical/electrochemical, mechanical, and biological stability under use-relevant conditions.
3. Wireless readout and power integration	Body loading, orientation, deformation, passive-array scaling, rigid electronics, packaging, and intermittent energy sources can constrain system operation.	Validate wireless links under realistic body-coupled conditions, improve scalable multi-channel readout, and demonstrate sustained energy delivery to the actual sensing, processing, and communication load. For light-exposed systems, benchmark PV or hybrid harvesting under representative illumination and body-worn conditions.	Reliable sensing, communication, and power delivery under intended operating conditions and realistic duty cycles, with harvested/stored energy matched to the actual workload where self-powered operation is claimed.
4. Integrated wearable-platform robustness	Fit, placement, slippage, re-donning, packaging, adhesion or washability, and technical failures can alter signals after component-level validation.	Evaluate complete wearable systems across repeated donning, movement, realistic task variation, environmental exposure, and repeated-use; characterize calibration retention and technical-failure modes.	Integrated platform maintains calibrated performance and acceptable usability across repeated wears and representative use conditions.
5. AI-enabled interpretation and multimodal robustness	User/session variability, calibration burden, missing channels or modalities, and distribution shift can reduce generalization despite high nominal model performance.	Develop failure-aware multimodal models, low-burden adaptation, channel/modality-loss tolerance, and evaluation across genuinely unseen users, sessions, tasks, devices, or environments.	Robust and interpretable performance on unseen users and conditions, with bounded calibration burden and graceful degradation when inputs become unreliable.
6. Controlled human and intended-user validation	Healthy-participant feasibility, acute evidence, or laboratory accuracy does not establish validity, reliability, or usefulness in the intended population.	Use appropriate reference systems, repeated-session designs, intended-user cohorts, and functionally or clinically meaningful outcomes matched to the claimed use.	Demonstrated validity and reliability of the integrated system for the intended measurement or functional outcome in relevant users or biological contexts.
7. Real-world and healthcare deployment	Calibration retention, charging/connectivity burden, missing data, maintenance, comfort, adherence, interoperability, workflow fit, privacy, and safety can become limiting outside controlled settings.	Conduct longitudinal home, community, or clinical-workflow studies that evaluate sustained data quality, adherence, maintenance burden, interoperability, and user/clinician experience.	Sustained operation with reliable data, acceptable user and system burden, interpretable outputs, and demonstrated compatibility with the intended workflow.
8. Application-dependent closed-loop personalization	Accurate offline sensing or prediction does not necessarily produce timely, useful, or appropriate feedback or intervention.	Instrument end-to-end loop latency, incorporate calibrated uncertainty and failure handling, co-design feedback with users or clinicians, and evaluate meaningful behavioural, functional, or clinical outcomes.	Closed-loop feedback or intervention demonstrates measurable benefit with appropriate latency, uncertainty handling, and user/clinician acceptability in the intended context.

## Data Availability

No new primary experimental data were generated. The quantitative information used in this review was extracted or derived from the cited literature, with supporting details provided in the Supplementary Materials.

## Supplementary Materials

The following supporting information can be downloaded at: https://www.mdpi.com/article/10.3390/s26216877/s1, Supplementary Note S1. Section-level literature sources and search documentation. Table S1. Literature sources, search dates, coverage windows, and limits for the six technical evidence streams. Supplementary Search Documentation S1A–S1F. Supplementary Note S2. Section 5 detailed evidence. Table S2. Detailed evidence records discussed in Section 5: wireless readout, radio frequency sensing, and power strategies (35 records). Supplementary Note S3. Figure 6 source definitions and evaluation context. Table S3. Source-reported endpoints and evaluation context for Figure 6. Supplementary Note S4. Figure 7 timing and resource definitions. Table S4. Timing provenance, boundaries and resource definitions supporting Figure 7. Supplementary Note S5. Exact paired simulation configurations for Figure 7b. Table S5. Twelve paired simulation-derived configurations from [221]. Supplementary Note S6. E1–E5 evidence contexts and standards/guidance crosswalk. Table S6. Conceptual crosswalk between the five complementary evidence contexts (E1–E5) and selected standards and guidance. Supplementary Note S7. Patent implementation and translational context. Table S7. Representative patent families providing implementation and translational context for wearable and body-interfaced sensing.
