# Supplementary material for: From Functional Nanomaterials to Trustworthy Wearable Sensing Systems: A Cross-Layer Review of Bioelectronic Interfaces, Wireless and Power Integration, Artificial Intelligence Inference/Interpretation, and Human-Centred Validation
Source: Sensors (Basel). 2026 Oct 30;26(21):6877. doi: 10.3390/s26216877 (PMC13766548; doi:10.3390/s26216877)
Supplement: Supplementary file 1 [file sensors-26-06877-s001.zip › sensors-4566320-supplementary.pdf]

Supplementary Materials

The search documentation describes literature identification for this structured critical review. It does not represent a Preferred Reporting Items for Systematic Reviews and Meta-Analyses (PRISMA) flow, an exhaustive systematic-review screening record, or a scoping-review protocol. Artificial intelligence (AI) assistance and author responsibility are described in Section 2.1 of the main manuscript.

Supplementary Note S1. Section-level literature sources and search documentation

Table S1. Literature sources, search dates, coverage windows, and limits for the six technical evidence streams.

Evidence stream	Information sources	Search date(s)	Coverage period	Limits
Sec. 3. Functional nanomaterial platforms	Web of Science Core Collection	1 Aug 2026	2015–2026	English; Article and Review Article.
Sec. 4. Bioelectronic interfaces	Web of Science Core Collection; Scopus	1–2 Aug 2026	2015–2026	English; Article and Review Article.
Sec. 5. Wireless readout, radio frequency (RF) sensing, and power	Scopus; Web of Science Core Collection; IEEE Xplore	6–7 Aug 2026	2015–2026	English; Article and Review Article.
Sec. 6. Integrated wearable platforms	Scopus; Web of Science Core Collection; PubMed	4 Aug 2026	2015–2026	English; Article and Review Article.
Sec. 7. AI-enabled interpretation, multimodal fusion, and feedback	Scopus; Web of Science Core Collection; IEEE Xplore; PubMed; Semantic Scholar; OpenAlex; arXiv	9 Aug 2026	2015–2026	English; Article, Review, and Conference/Proceedings Paper.
Sec. 8. Application-level evidence and translational gaps	Web of Science Core Collection; Scopus; PubMed; IEEE Xplore; Google Scholar	4 Aug 2026	2015–2026	English; Article and Review Article.

The publication-type limits describe the documented searches, rather than an exhaustive restriction on the publication types cited in the final synthesis.

Supplementary Search Documentation S1A–S1F

S1A. Section 3 – Functional nanomaterial platforms. Web of Science Core Collection: ("wearable sensor*" OR "wearable biosensor*" OR "wearable electronic*" OR "wearable device*" OR "electronic skin" OR "e-skin" OR "flexible sensor*"). Targeted supplementary searches (Web of Science Core Collection): MXene: (("wearable sensor*" OR "wearable biosensor*" OR "wearable electronic*" OR "wearable device*" OR "electronic skin" OR "e-skin" OR "flexible sensor*") AND (MXene* OR Ti3C2 OR Ti3C2Tx OR "transition metal carbide*" OR "transition-metal carbide*" OR "2D carbide*")); Graphene: (("wearable sensor*" OR "wearable biosensor*" OR "wearable electronic*" OR "wearable device*" OR "electronic skin" OR "e-skin" OR "flexible sensor*") AND (graphene* OR "graphene oxide" OR "reduced graphene oxide" OR rGO)); MOF: (("wearable sensor*" OR "wearable biosensor*" OR "wearable electronic*" OR "wearable device*" OR "electronic skin" OR "e-skin" OR "flexible sensor*") AND (MOF OR MOFs OR "metal-organic framework*" OR "metal organic framework*")); Hydrogel: (("wearable sensor*" OR "wearable biosensor*" OR "wearable electronic*" OR "wearable device*" OR "electronic skin" OR "e-skin" OR "flexible sensor*") AND (hydrogel* OR "conductive hydrogel*" OR "ionic hydrogel*" OR "self-healing hydrogel*" OR organohydrogel*)).

S1B. Section 4 – Bioelectronic interfaces. Web of Science Core Collection and Scopus: ("functional nanomaterial*" OR graphene OR "carbon nanotube*" OR CNT OR MXene* OR "conductive polymer*" OR "PEDOT:PSS" OR "PEDOT PSS" OR hydrogel* OR nanofiber* OR nanocomposite* OR nanoparticle* OR "nanoparticle coating*" OR "conductive textile*") AND ("flexible sensor*" OR "wearable sensor*" OR "implantable sensor*" OR "bioelectronic interface*" OR "soft electrode*" OR "epidermal electrode*" OR "implantable electrode*" OR "neural interface*") AND (electrophysiolog* OR electrochemical* OR "optical sensing" OR optoelectronic* OR optogenetic* OR biosignal* OR ECG OR EMG OR EEG OR "neural recording" OR "neural sensing" OR transduction OR "device architecture*"). Targeted optical/optogenetic supplement (Web of Science Core Collection and Scopus): (nanomaterial* OR nanoparticle* OR nanocomposite* OR nanofiber* OR graphene OR "carbon nanotube*" OR CNT OR MXene* OR "conductive polymer*" OR "nanostructured hydrogel*") AND ("optical fiber*" OR "hydrogel fiber*" OR "bioelectronic fiber*" OR "neural interface*") AND (optogenetic* OR "optical stimulation" OR photostimulation) AND ("peripheral nerve*" OR neural OR nerve*).

S1C. Section 5 – Wireless readout, radio-frequency (RF) sensing, and power. Scopus, Web of Science Core Collection, and IEEE Xplore: Block 1: (flexible OR stretchable OR wearable OR textile OR epidermal OR "skin-mounted" OR conformal OR implantable) AND (antenna* OR resonator* OR "split ring" OR "split-ring" OR "frequency selective surface" OR metamaterial* OR microwave OR "radio frequency" OR chipless OR RFID OR "LC resonan*" OR "passive wireless") AND (sensor* OR sensing OR interrogat*) AND (graphene OR "carbon nanotube*" OR CNT OR MXene OR nanomaterial* OR nanocomposite* OR nanoparticle* OR nanowire* OR nanofib* OR nanotube* OR "quantum dot*" OR "two-dimensional material*" OR "2D material*" OR nanostructur* OR nanosheet* OR "silver nanowire*" OR MoS2 OR "transition metal dichalcogenide*"). Block 2: (wearable OR flexible OR epidermal OR textile OR "electronic skin" OR "e-skin") AND ("Bluetooth low energy" OR BLE OR NFC OR "near-field communication" OR RFID OR "body area network" OR "human body communication" OR "body-coupled" OR battery-free OR batteryless OR "readout circuit*" OR "front-end" OR multiplex*) AND (sensor* OR sensing OR monitor*) AND (graphene OR "carbon nanotube*" OR CNT OR MXene OR nanomaterial* OR nanocomposite* OR nanoparticle* OR nanowire* OR nanofib* OR nanotube* OR "quantum dot*" OR "two-dimensional material*" OR "2D material*" OR nanostructur* OR nanosheet* OR "silver nanowire*" OR MoS2 OR "transition metal dichalcogenide*"). Block 3: (wearable OR flexible OR "on-body" OR textile OR epidermal OR stretchable) AND (triboelectric OR piezoelectric OR photovoltaic OR "solar cell*" OR thermoelectric OR "biofuel cell*" OR "energy harvest*" OR "self-powered") AND (sensor* OR sensing OR "power source*") AND (graphene OR "carbon nanotube*" OR CNT OR MXene OR nanomaterial* OR nanocomposite* OR nanoparticle* OR nanowire* OR nanofib* OR nanotube* OR "quantum dot*" OR "two-dimensional material*" OR "2D material*" OR nanostructur* OR nanosheet* OR "silver nanowire*" OR MoS2 OR "transition metal dichalcogenide*") AND ("power management" OR "boost convert*" OR "duty cycl*" OR microcontroller OR MCU OR bluetooth OR BLE OR "wireless transmission" OR "energy budget" OR supercapacitor OR "self-charging" OR "power a load" OR "system-level" OR "fully integrated" OR "energy storage"). Block 4: (wearable OR flexible OR "on-body" OR "body-worn" OR "skin-mounted") AND (antenna* OR "radio frequency" OR microwave OR resonat* OR wireless) AND (detun* OR "body effect*" OR "specific absorption rate" OR encapsulat* OR washab* OR "motion artifact*" OR "motion artefact*" OR breathab* OR "long-term wear" OR "clinical translation") AND (graphene OR "carbon nanotube*" OR CNT OR MXene OR nanomaterial* OR nanocomposite* OR nanoparticle* OR nanowire* OR nanofib* OR nanotube* OR "quantum dot*" OR "two-dimensional material*" OR "2D material*" OR nanostructur* OR nanosheet* OR "silver nanowire*" OR MoS2 OR "transition metal dichalcogenide*"). Block 5: (graphene OR "carbon nanotube*" OR MXene OR nanocomposite* OR nanoparticle* OR nanotube* OR "graphene oxide" OR "two-dimensional material*" OR nanomaterial*) AND ("microwave resonator*" OR "planar resonator*" OR "split ring resonator*" OR "cavity perturbation" OR "dielectric resonator" OR "coplanar waveguide" OR "microwave characteri*") AND (permittivity OR conductivity OR "dielectric propert*" OR "complex permittivity" OR sensing OR characteri*). Block 6: (wearable OR flexible OR epidermal OR skin OR sweat OR saliva OR "interstitial fluid" OR noninvasive OR non-invasive OR "point-of-care") AND ("split ring resonator*" OR "microwave resonator*" OR "planar resonator*" OR "RF resonator*" OR "LC resonan*" OR "interdigital capacitor*" OR "microwave biosensor*" OR "microwave sensor*") AND (glucose OR lactate OR cortisol OR biomarker* OR "molecularly imprinted" OR aptamer* OR antibod* OR enzymat* OR "glucose oxidase" OR "boronic acid" OR biorecognition OR selectivit* OR specificit*). Block 7: (nanocomposite* OR "carbon nanotube*" OR graphene OR "graphene nanoplatelet*" OR "carbon black" OR MXene OR "conductive filler*" OR buckypaper) AND ("cavity perturbation" OR "resonant cavity" OR "dielectric resonator" OR "split ring resonator*" OR "quality factor" OR "loaded Q" OR "resonant frequency shift") AND (percolation OR "percolation threshold" OR "effective medium" OR "Maxwell-Garnett" OR Bruggeman OR Lichtenecker OR "volume fraction" OR "filler loading"). Four additional records were added through targeted supplementary identification rather than the documented Section 5 database searches; these records are marked with a dagger (†) in Table S2.

S1D. Section 6 – Integrated wearable platforms. Scopus, Web of Science Core Collection, and PubMed: (("wearable sensor*" OR "smart textile*" OR "smart garment*" OR "smart sleeve*" OR "smart brace*" OR "smart glove*" OR "pressure insole*" OR smartwatch OR IMU) AND (rehabilitation OR gait OR "human motion" OR "range of motion" OR "hand function" OR haptic OR tactile) AND (validation OR "clinical validation" OR "healthy subject*" OR patient* OR "motion capture" OR "force plate" OR "ground truth")).

S1E. Section 7 – AI-enabled interpretation, multimodal fusion, and feedback. Scopus; Web of Science Core Collection; IEEE Xplore; PubMed; Semantic Scholar; OpenAlex; arXiv: (("wearable sensor*" OR "textile sensor*" OR "smart textile*" OR "smart garment*" OR "smart glove*" OR "tactile glove*" OR "pressure insole*" OR IMU OR "inertial measurement unit" OR sEMG OR "surface electromyograph*") AND ("machine learning" OR "deep learning" OR "sensor fusion" OR "multimodal fusion" OR CNN OR "convolutional neural network*" OR LSTM OR "recurrent neural network*" OR "transformer model*" OR "attention mechanism*" OR "self-attention" OR "edge computing" OR "real-time feedback" OR personalization OR personalisation OR "domain adaptation" OR "transfer learning" OR "digital twin") AND ("human motion" OR rehabilitation OR gait OR tactile OR haptic OR "human-machine interface" OR "human-computer interaction" OR "intent estimation")).

S1F. Section 8 – Application-level evidence and translational gaps. ("wearable sensor*" OR "wearable biosensor*" OR "flexible sensor*" OR "electronic skin" OR "smart wearable") AND (graphene OR MXene OR "carbon nanotube*" OR hydrogel OR "conductive polymer*" OR nanomaterial*) AND (physiological OR biochemical OR optical OR rehabilitation OR telemedicine OR "digital health" OR "human-machine interaction").

Supplementary Note S2. Section 5 detailed evidence

Table S2. Detailed evidence records discussed in Section 5: wireless readout, radio frequency sensing, and power strategies (35 records).

Reference	Section	Strategy/mechanism	Key reported figure	Validation context/evidence type
[126]	5.1	Graphene FET harmonic transponder (frequency multiplication)	1.38 V per pH Dirac-point shift; return tone at a multiplied frequency	Single biased transistor measured; full transponder simulated only; no wireless link demonstrated
[121]	5.1	Chipless LC, Au-coated TiO_2 nanowires in silicone	260 kPa modulus at 40% strain; read through 50% strain cycles with coils ~1 cm apart	Ex vivo porcine bladder; several cm quoted only as an upper bound
[130]	5.1	Chip-free battery-free printed tag, detuned RFID load modulation	Tag 76.2 MHz vs 13.56 MHz carrier, 50.0 MHz at 50% strain, Q 12.9→1.2; readout gain near zero beyond 20 mm vertical separation; approximately 25 mm lateral antenna misalignment tolerated	Bench range sweep; 5 nodes, pulse/respiration/movement, 2 participants (both authors)
[129]	5.1	Ag-nanofiber network epidermal antenna/NFC coil	Transmission efficiency 35%→15% at 10 MHz under 100% strain; FM link at 1 m	On-skin bench
[124]	5.1	Nanostrip metamaterial microstrip antenna + glucose oxidase	Calculated limit of detection: 0.1 nM, 0.1 nM–10 mM range in buffer; 2× sensitivity of flat film	On-skin test separated two simulated-sweat levels; read over a cable to a VNA, not wirelessly
[125]	5.1	Freestanding GaN surface-acoustic-wave chipless e-skin	Strain limit of detection 0.048% (bench); link ~14 mm axial, ~7.5 mm lateral	Daytime wrist-pulse monitoring over 7 days (~17 h/day); separate on-body sweat Na^+ sensing
[21]	5.1	AgNW antennas on porous SEBS + passive Schottky-diode multiplier	Zero-power cough and mask-displacement reporting over a fixed 0.5 m link	One wearer
[127]	5.1	MWCNT/PDMS frequency-multiplexed resonator array	4 resonators 15 MHz apart; ~1 Pa LOD; 17.49 MHz kPa^{-1} below 4 kPa, 0.45 MHz kPa^{-1} above 20 kPa	98.5% position accuracy from a trained classifier on held-out data — peaks overlap, not directly readable
[122]	5.1	AgNP/chlorhexidine hydrogel + split-ring resonator	H_2S 2–30 µM in artificial saliva, limit of detection 1.2 µM calculated; simultaneous drug release in vitro	Tooth-mounted patch; collected saliva separated 4 periodontitis patients from 3 healthy participants
[128]	5.1	Nonlinear parity–time-symmetric reader, MWCNT/PDMS force array	Coupling-robust while in the PT-symmetric phase, below ~6 mm; 3D force via decoupling network	Bench
[123]	5.1	PEDOT:PSS/graphene 110 nm film on UHF RFID tag	1.568 dBm/°C on a muscle-equivalent phantom; 0.437 °C mean error versus an infrared reference	Trained on 35 of 50 participants; error reported for the 15 held-out participants
[133]	5.2	Batteryless crystal-free SoC + CNT supercapacitor	24 µW per SoC; 2.025 mm^2 in 0.18 µm CMOS	Single chip closed a 457.5 MHz link at 1 m; two synchronized nodes recorded EMG from a human arm via fine-wire electrodes (no distance reported)
[135]	5.2	Intrinsically stretchable polymer diodes	13.56 MHz rectification	Stretchable tag demonstrator
[136]	5.2	CNT ion-selective FET + bendable front-end + BLE SoC	4.516 µA/decade for ammonium over 0.1–100 mM	Five-device proof of concept in artificial sweat; matched a benchtop analyzer at 10 kΩ feedback resistance but saturated at 82 kΩ
[6]	5.2	Dielectro-elastic substrate for strain-invariant RF	Resonance shift ~5 MHz; comparison range 50–1250 MHz from other published elastomer-supported antenna designs (not matched); wireless telemetry up to 30 m under tested deformation	Skin-interfaced monitors
[134]	5.2	CNT-fibre sweat patch + custom 4 × 4 mm SoC	Minimum average power of 33 µW at a 1:60 RF duty cycle—analog measured, digital simulated	Forehead-worn exercise monitoring of K^+ and lactate; NFC powering and data

Reference	Section	Strategy/mechanism	Key reported figure	Validation context/evidence type
[137]	5.2	Wafer-scale trilayer MoS_2 active array on polyimide	51 pixels mm^{-2}; ~20 ns multiplexing time constant for the array plus its external controller	ECoG in vitro; multiscale recording in living mice
[140]	5.3	Atomically thin MoS_2 heterojunction rectenna	10 GHz cutoff; up to 250 mV at ~2.5 cm, zero external bias, ~2 mW available at the rectenna	Signal-generator-driven source; ambient harvesting identified as future work
[7]	5.3	Lactate biofuel cell e-skin (0D–3D nanomaterial integration)	Results: up to 3.6 mW cm^{-2} in ex vivo sweat from four donors (abstract/discussion: 3.5 mW cm^{-2}); ~60 h charge–discharge in vitro with fuel replenishment	Drives multiplexed sensing and duty-cycled BLE on-body in three volunteers
[142]	5.3	3D-printed MXene triboelectric generator on SEBS	816.6 mW m^{-2} instantaneous peak into a 100 MΩ load under linear-motor actuation (8 Hz, 19.9 N)	Radial-artery pulse monitoring in one volunteer; operation depends on manual recharging of the storage capacitor
[145]	5.3	Ultra-wideband triboelectric ultrasonic film	1.2 V, 150 µA through 10 mm of excised porcine tissue, probe driven at 100 Vpp; telemetry to 4 cm	Ex vivo tissue and water tank; no in vivo demonstration
[141]	5.3	All-fabric 5.8 GHz rectenna glove	Powers yarn bend sensors at distances up to 1 m from a 30 dBm laboratory source and horn antenna; 98.75% object classification	On-hand demonstration; 4 cm is the magnetic-coupling limit cited by the authors, not a measured range
[147]	5.3	3D-printed stretchable microsystem, wirelessly charged Na-ion microbatteries	0.96 mAh cm^{-2} (8L+4L− stack); 70% retention after 1500 cycles (2L+1L− cell); 34.6% discharge-energy/receiving-coil-output-energy ratio (2L+1L− cell)	Simulated sweat; glucose detected down to 0.5 mM; 1000 stretch cycles at 50% strain
[146]	5.3	Battery-free NFC epidermal platform with microfluidics	1.2 cm across, 3 mm thick; Prussian-blue and rGO electrodes; reported MARD of 13.8% for blood-glucose estimates based on sweat glucose scaled 120-fold versus fingertip blood-glucose meter measurements	Glucose evaluated in three healthy volunteers against fingertip blood-glucose meter measurements; uric acid assessed separately in three volunteers against serum uric acid using a commercial meter
[143]	5.3	Interface-engineered Ag_2Se thermoelectric film	Power factor 3328 ± 332 µW m^{-1} K^{-2} measured; ZT 1.1 at 341 K calculated; five-leg generator gave 4.25 µW measured at ΔT = 35 K, 85 W m^{-2} in the Results, normalized to total thermoelectric-leg cross-section; abstract/conclusion: 81 W m^{-2} (unresolved reporting discrepancy)	A hot-water power demonstration used a boost converter for ~20 mV thermoelectric input; body-heat sensing was tested separately
[148]	5.4	Omniphobic silk-fibroin/MWCNT power-receiving coils	Device operation after 50 detergent-free machine-wash cycles; breathability retained after spray silanization	Sewn onto ordinary fabric
[149]	5.4	Textile triboelectric pulse sensor + ML blood-pressure model	Bench: 23.3 dB SNR, 40 ms sensor response; reported mean relative blood-pressure deviations versus a commercial cuff: 2.9% (systolic), 1.2% (diastolic)	One participant; operation under movement and moisture shown qualitatively (no dedicated robustness metric)
[152]	5.4	Printed nanomembrane strain sensors on an inductive stent	Passive, no battery; 2.5 cm radial and 1.0 cm axial in air; 1.5 cm radial and 0.5 cm axial in saline; signal lost at 90° rotation	Silicone artery phantom; ex vivo ovine coronary
[150]	5.4	Screen-printed AgNW wet ECG electrodes + antenna-on-package	Per-electrode conductive-metal mass: printed AgNW 0.86 ± 0.05 mg, commercial 227.72 ± 2.48 mg (printed ≈1/265); 142 m wireless range calculated, not measured	Blinded ECG-feature review (20 volunteers); one-volunteer wear test: normalized R-peak value fell ~15% at 20 h (printed), ~30% at 16 h (commercial), and ~35% for both at 24 h; printed AgNW layer delaminated in a separate 24 h artificial-sweat test
[151]	5.4	Battery-free permeable wound bandage, sensing + iontophoresis	Iontophoretic delivery in vitro; accelerated healing in infected diabetic rats; reported glucose, pH and temperature accuracy scores >80% against commercial references (25 plotted pairs per output)	Human sensing: 10 patients with mixed wounds; six assessed at dressing-change visits over two weeks, four assessed once

Reference	Section	Strategy/mechanism	Key reported figure	Validation context/evidence type
[153]	5.4	Battery-free CNT throat patch, NFC over 3–4 cm	84.7% on five fixed sentences; 90.2% on six emotion classes	Four-subject leave-one-subject-out evaluation: data from three subjects used for training, with one subject held out per fold (79.4–81.6% text accuracy); two additional subjects tested separately
[131]†	5.1	Passive LC tag for on-skin hydration-related dielectric sensing	Five participants; dry, wet, lotion-treated and Vaseline-treated forearm skin; five repeats per condition (100 datasets). Inductively coupled reader with external VNA over 50–200 MHz; reader–tag metallization separation approximately 0.8 mm	Controlled resting-arm tests at 23 °C; quantitative hydration calibration against an independent reference and motion-tolerant portable readout remain future work
[132]†	5.1	Battery-free NFC skin-moisture patch with resistive electrode readout	18 mm coil at 13.56 MHz compared with 27 and 36 mm coils; bending and water-immersion link tests; electrode-resistance signal digitized by the NFC chip. Separate droplet and skin demonstrations include before/after-shower readings over seven days	External reader required; participant count is not stated. Absolute hydration accuracy and simultaneous on-body link stability across the tested conditions were not established
[144]†	5.3	$CsPbI_2Br$-decorated PVDF piezoelectric nanogenerator yarn	8.3 V, 1.91 µA under actuator pressing; 281.0 µW cm^{-3} peak into 300 MΩ at 16 kPa, volume-normalized to the nanofiber layer; 85.6 µW cm^{-3} instantaneous and 10.6 µW cm^{-3} average from capacitor charging	A 1.1 µF capacitor was charged to 1.8 V in 50 s via a bridge rectifier and discharged to light an LED—not a sensing node; 19,200 cycles were completed three months after fabrication
[53]†	5.3	Solid-state Zn/MnO_2 fibre battery, graphene-oxide-embedded poly(vinyl alcohol) hydrogel electrolyte	Coin-cell electrochemical tests: stable voltage response beyond 500 h; 98.0% capacity retention after 1000 cycles at 0.3 A g^{-1}	Powers simultaneous heart-rate, temperature, humidity, and altitude monitoring from an on-body e-textile

Note: Numerical values are reproduced as reported in the cited articles and were checked against the available full texts. Calculated, simulated, or proxy-condition results are identified explicitly. Records marked † were added through targeted supplementary identification (4 of 35); the remaining 31 records came from the documented Section 5 database searches. Operating inputs, normalization bases, and validation settings differ across records, so the reported values provide study-specific evidence rather than a common performance ranking.

Abbreviations: 3D, three-dimensional; AgNP, silver nanoparticle; AgNW, silver nanowire; BLE, Bluetooth Low Energy; CMOS, complementary metal–oxide–semiconductor; CNT, carbon nanotube; ECG, electrocardiography; ECoG, electrocorticography; EMG, electromyography; FET, field-effect transistor; FM, frequency modulation; GaN, gallium nitride; LC, inductor-capacitor; LED, light-emitting diode; LOD, limit of detection; MARD, mean absolute relative difference; ML, machine learning; MWCNT, multiwalled carbon nanotube; MXene, a family of two-dimensional transition-metal carbides and nitrides; NFC, near-field communication; PANI, polyaniline; PDMS, polydimethylsiloxane; PEDOT:PSS, poly(3,4-ethylenedioxythiophene):polystyrene sulfonate; PT, parity–time; PVA, poly(vinyl alcohol); PVDF, poly(vinylidene fluoride); RF, radio frequency; RFID, radio frequency identification; rGO, reduced graphene oxide; SEBS, styrene-ethylene-butylene-styrene; SNR, signal-to-noise ratio; SoC, system-on-chip; UHF, ultra-high frequency; VNA, vector network analyzer; Vpp, peak-to-peak voltage; ZT, dimensionless thermoelectric figure of merit.

Supplementary Note S3. Figure 6 source definitions and evaluation context

Figure 6 compares source-reported summary endpoints within their stated evaluation settings. Table S3 identifies each endpoint, its source locator and the meaning of its uncertainty. Reference numbers refer to the main manuscript bibliography. CV denotes cross-validation; CNN, convolutional neural network; RMSE, root mean square error; SD, standard deviation.

Table S3. Source-reported endpoints and evaluation context for Figure 6.

Panel	Reference/source locator	Source-reported endpoint	Evaluation context	Uncertainty/interpretation
6a	[9], Results and Table 6	10-fold CV: 99.85%; leave-one-subject-out: 85.1%. Observed gap: 14.75 percentage points.	Same CNN-2C-1D configuration, vector magnitude and 50-sample sliding window on MHEALTH.	The observed gap does not isolate a causal leakage effect.
6b	[201], Table 2, Kalman-refined Average column	Small hand: self-calibration 3.91°, non-calibration 4.69°. Large hand: self-calibration 4.16°, non-calibration 7.14°.	Four distinct participants, one per hand-size/calibration cell. Each mean absolute error averages ten joint degrees of freedom.	No across-participant uncertainty estimate. The separate 13-participant bone-length dataset is not the sample size of this comparison.
6c	[22], Figure 3 and Results	Individualized RMSE: 2.81 ± 0.75°; adaptive RMSE: 5.52 ± 2.10°; generalized RMSE: >10°.	Long short-term memory models evaluated on the common last-20% test segment of one participant.	SD across joint-angle outputs; exact output membership and weighting are not reported. It is not participant-level uncertainty or the standard errors across 32 gait cycles in separate Figure 4 trajectory bands. >10° is a strict lower bound; no rounded ratio or propagated ratio uncertainty.
6d	[204], Table 4, vemg2pose regression row	Held-out users: 12.2 ± 1.3°; held-out stages: 15.2 ± 1.6°; held-out users + stages: 15.8 ± 1.4°.	Mean absolute angular error on three distinct test sets: unseen users with training stages; unseen stages with training users; both unseen. A stage is a category of hand movements.	SD across users. Different generalization axes, not a paired deterioration experiment. Tracking with a known initial pose is a separate task.

Supplementary Note S4. Figure 7 timing and resource definitions

Figure 7 distinguishes reported empirical timing, tool estimates and simulation-derived inference timing. Inference duration alone does not establish sensing-window acquisition, preprocessing, communication, feedback, actuation or human-response delay. Table S4 preserves source-specific boundaries and hardware; Table S5 contains the exact paired simulation configurations. These quantities do not support a universal 100 ms threshold or cross-study latency ranking. Model-file size, deployed flash, random-access memory (RAM) and source-reported model memory remain distinct. Here, float32 denotes 32-bit floating point; INT8, 8-bit integer quantization; PDF, portable document format; PULP, Parallel Ultra-Low Power; KAN, Kolmogorov–Arnold network; HAR, human activity recognition; SRAM, static RAM. References refer to the main manuscript bibliography.

Table S4. Timing provenance, boundaries and resource definitions supporting Figure 7.

Reference	Quantity shown	Timing boundary and provenance	Hardware/compute context	Resource definitions and necessary limits
[218]	90 ms; 110 ms; 0.6 s (600 ms)	Reported tactile/electrical feedback-path responses; separately, obstacle detection to initial flight-path change.	Skin-interfaced controller and closed-loop drone system.	The 0.6 s endpoint is initial path change, not completion of correction. No interval or independent timing sample size inferred. Source: PDF p. 11.
[219]	769 ms float32; 80 ms INT8	Edge Impulse/EON Compiler tool estimates for model inference; not a measured full loop.	nRF52840 target, Cortex-M4F at 64 MHz; stride-length model.	Preprocessing excluded. Static-buffer test had no live inertial input. Estimated flash: 284.5/91.6 kB; RAM: 31.9/13.6 kB (float32/INT8). These are not model-file sizes. Source: PDF pp. 7–8, Table 5 p. 10, Discussion p. 11.
[220]	11.67 ± 0.67 ms	Reported offline Python pipeline: signal reading, preprocessing and output, over ten timing experiments.	Tightly coupled convolutional transformer knee-angle prediction; timing hardware not established.	The ± statistic is undefined; it is not identified as a standard deviation or confidence interval. Input-window/acquisition delay is not fully specified; no deployed exoskeleton loop. Source: PDF pp. 1, 8.
[222]	97.93 ms	Reported mean multiscale attention patching encoder network (sMAPEN) inference time; not acquisition-to-action latency.	Raspberry Pi 4B: quad-core Cortex-A72, 1.5 GHz, 4 GB RAM.	Table 3 averages over 40 subjects. 100 ms feature windows and 0.5 ms stride are separate from model timing; no timing error bar reported. Source: PDF pp. 3, 7–8, Table 3 p. 12.

Reference	Quantity shown	Timing boundary and provenance	Hardware/compute context	Resource definitions and necessary limits
[221]	Twelve pairs in Table S5	Simulation-derived inference latency from GVSOC cycles; no measured deployment implied.	PULP/Quentin assumptions: 205.1 MHz, 0.54 V; power-gated after inference.	Reported memory/latency/configuration pairs are kept together. Independent extrema do not define a jointly achievable rectangle. No timing error bars supplied. Source: PDF pp. 10–11, 17–18, Table 5 p. 24.
[224]	Resource-only example; not plotted in Figure 7a	Model-file size, deployed flash and RAM are separate resource quantities.	DeepConv LSTM (long short-term memory); Arduino Nano 33 BLE Sense Rev2/Edge Impulse; 5 s data windows.	Model-file size: 136.51 KB; deployed flash: 189.6 KB; RAM: 29.1 KB. No verified complete-loop timing is established. Source: PDF pp. 12, 14–15, Figure 19.
[223]	4.1 ms per window	Reported on-device INT8 TinyKAN-HAR model timing; not sensing-to-action latency.	Cortex-M4F-class microcontroller at 80 MHz; 256 kB flash, 64 kB static RAM (SRAM).	Network plus runtime flash 145 kB; peak SRAM 26 kB. No latency dispersion reported; energy elsewhere is estimated. Source: PDF pp. 35–36, Table 10.

Supplementary Note S5. Exact paired simulation configurations for Figure 7b

The twelve configurations in Table S5 retain the dataset-specific score, reported model memory, energy and inference latency from Table 5 of [221] (PDF p. 24). Latency and energy are simulation-derived under Quentin assumptions of 205.1 MHz and 0.54 V, with power gating after inference; they are not measured deployment results. Min, Max-5% and Max are source configuration labels, and score metrics differ across datasets. Pairings are retained within each row. No interpolation, feasible rectangle, cross-dataset ranking or uncertainty is inferred. Values retain the source units; kB is not relabeled KiB. Timing/resource boundaries are defined in Table S4.

Table S5. Twelve paired simulation-derived configurations from [221].

Dataset	Configuration	Score metric	Score (%)	Reported memory (kB)	Energy (µJ)	Latency (ms)
UniMiB-SHAR	Min	balanced accuracy	26.24	0.41	0.34	0.09
UniMiB-SHAR	Max-5%	balanced accuracy	81.56	9.28	29.33	7.7
UniMiB-SHAR	Max	balanced accuracy	86.24	23.17	61.59	16.2
UCI HAPT	Min	accuracy	44.53	0.42	0.44	0.12
UCI HAPT	Max-5%	accuracy	83.18	4.6	30.52	8.01
UCI HAPT	Max	accuracy	85.63	7.54	23.27	6.11
WISDM	Min	F1	67.6	0.22	0.3	0.08
WISDM	Max-5%	F1	94.74	1.27	4.09	1.07
WISDM	Max	F1	98.9	6.22	15.94	4.19
WALK	Min	balanced accuracy	78.13	0.05	0.03	0.009
WALK	Max-5%	balanced accuracy	91.81	0.18	0.05	0.016
WALK	Max	balanced accuracy	95.74	1.65	0.67	0.18

Supplementary Note S6. E1–E5 evidence contexts and standards/guidance crosswalk

The five complementary evidence contexts (E1–E5) distinguish the evaluation questions associated with material/component function, integrated-device operation, controlled human measurement, intended-user relevance, and everyday or longitudinal use. A study may support multiple contexts; progression is neither mandatory nor linear, and E5 is not inherently better than E3 or E4. They are not consensus standards, regulatory stages, maturity levels or ordinal quality scores. The selected external frameworks overlap particular evidence questions and do not define E1–E5. Table S6 is a conceptual crosswalk and does not establish regulatory applicability, conformity, compliance, certification or equivalence.

Applicability depends on intended use, device classification, jurisdiction and study design. ISO/IEC interpretation uses official public scope and edition information; full standards were not reviewed. FDA digital health technologies (DHT) guidance was reviewed in full. References [186–190] are in the main manuscript bibliography. Risk management and usability can span multiple contexts. ISO denotes International Organization for Standardization; IEC, International Electrotechnical Commission; FDA, U.S. Food and Drug Administration.

Table S6. Conceptual crosswalk between the five complementary evidence contexts (E1–E5) and selected standards and guidance.

Evidence context	Assessment focus	Related standards/guidance	Relevance and boundary
E1	Material/component function under specified physical/environmental conditions.	FDA DHT technical/measurement verification, where applicable [190]; cross-cutting ISO 14971 risk management [186].	Partial/conceptual. DHT guidance concerns clinical investigations; it does not define material test methods or E1 as a regulatory stage.
E2	Function after integration into the complete wearable/device architecture.	IEC 60601-1 basic safety/essential performance, where applicable [187]; IEC 62366-1 usability engineering [188]; ISO 14971 risk management [186].	Partial/application-specific. These sources do not validate all adhesion, washing, wireless or re-donning issues, or define E2 as a maturity level.
E3	The claimed measurement property in controlled human testing.	FDA DHT measurement validation [190]; ISO 14155 for applicable medical-device clinical investigations [189].	Partial/study-design-specific. Reliability is not accuracy. Not every controlled human validation study is automatically an applicable ISO 14155 clinical investigation.
E4	Interpretability and usability in the intended population and use context.	IEC 62366-1 usability engineering [188]; ISO 14155 clinical-investigation principles, where applicable [189]; FDA DHT intended-population considerations [190].	Overlapping/application-specific. Usability, clinical validity and clinical benefit are distinct. Target-population participation alone does not prove clinical benefit.
E5	Acceptable operation under everyday and/or longitudinal use.	ISO 14971 lifecycle risk management [186]; FDA DHT remote-operation, data-integrity and operational considerations [190].	Partial/cross-cutting. Real-world setting and duration are separate attributes. Real-world operation, longitudinal follow-up, post-market surveillance and clinical benefit are not equivalent.

Supplementary Note S7. Patent implementation and translational context

Patent publications provide architecture, integration, manufacturing and implementation/translational-pathway context. This illustrative, non-exhaustive set uses one representative document per family and broadens implementation categories and institutional representation. National counterparts and continuations are family-deduplicated. P.S. is a named inventor on the five author-affiliated families [273–277]; no current manuscript author was identified as an inventor in the selected publications for the five independent examples [278–282]. Patent disclosure does not establish peer-reviewed performance validation, clinical effectiveness, commercialization, market adoption, commercial success, regulatory approval or superiority. Described embodiments are not assumed to equal granted claim scope. Applicants/assignees and qualified priority dates refer to the selected publication/family record, not verified current ownership or claim-specific priority. No patent-prevalence or technology-quality inference is made. “No current manuscript author identified as inventor” refers only to the screened inventor list and does not establish absence of every institutional or financial relationship. Reference numbers refer to the main manuscript bibliography.

Table S7. Representative patent families providing implementation and translational context for wearable and body-interfaced sensing.

Representative patent family/publication	Applicant/assignee	Implementation focus	Evidence boundary	Author relationship

Representative patent family/publication	Applicant/assignee	Implementation focus	Evidence boundary	Author relationship
[273] US9494474B2<br>Priority: 3 Apr 2013, US61/808,096.	Assignee: Texavie Technologies Inc.	Textile/nanofiber strain sensing and manufacturing; core-shell fibres and textile transduction.	Patent disclosure, not independent performance validation.	P.S. is a named inventor.
[274] US20210255694A1<br>Priority: 19 Apr 2018, US62/660,168.	Applicant/assignee: Texavie Technologies Inc.	Wearable deformation/topography inference; deformation signals mapped to relative body-part positions.	Does not independently validate estimation accuracy, clinical utility or rehabilitation benefit.	P.S. is a named inventor.
[275] WO2024092368A1<br>Priority: 4 Nov 2022, US63/422,867.	Applicant: Texavie Technologies Inc.	Stretchable textile wearable integration; yarn sensors, computing and body-metric inference.	Does not establish clinical utility, ML generalization or service lifetime.	P.S. is a named inventor.
[276] CA3153167A1<br>PCT/CA2020/051202; WO2021042215A1. Priority: 6 Sep 2019, US62/897,182.	Applicant: The University of British Columbia.	Body-interfaced optical/near-infrared spectroscopy architecture, particularly spinal-cord monitoring.	Not demonstrated routine noninvasive everyday skin-worn monitoring.	P.S. is a named inventor.
[277] WO2022126279A1<br>Priority: 18 Dec 2020, US63/199,326.	Applicant: Texavie Technologies Inc.	Adjacent thermal/enabling-material technology; insulating substrate manufacture.	Not a sensing mechanism or evidence of validated sensor-packaging performance.	P.S. is a named inventor.
[278] US10441185B2<br>Continuity chain includes 16 Dec 2009; selected branch also claims 10 Jun 2011 provisional. Not priority for every claim/feature.	Assignee: The Board of Trustees of the University of Illinois.	Epidermal electronics; compliant circuits, electrophysiological arrays and serpentine interconnects.	Does not independently establish universal skin safety, long-term wear reliability or validation of every embodiment.	No current manuscript author identified as inventor.
[279] WO2017218878A1<br>Priority: 17 Jun 2016, US62/351,734; additional provisional: 15 Nov 2016, US62/422,536.	Applicants: The Board of Trustees of the University of Illinois; Northwestern University.	Wearable microfluidics/biochemical sensing; soft skin-mounted biofluid collection, storage, temporal sampling and sensing.	Does not establish diagnosis, sweat–blood equivalence, generalized clinical biomarker validity or universal lifetime.	No current manuscript author identified as inventor.
[280] WO2017185050A1<br>Selected WO: 21 Apr 2016, US62/325,901; 14 Feb 2017, US62/458,795. Extended U.S. lineage reaches 30 Jan 2012; not all-feature/claim priority.	Applicant: Sensoria, Inc.	Garment/footwear sensor assembly; carrier layers, conductive traces, sensors, connectors and acquisition electronics.	Does not establish wash lifetime, manufacturing yield, production cost, accuracy or treatment benefit.	No current manuscript author identified as inventor.

Representative patent family/publication	Applicant/assignee	Implementation focus	Evidence boundary	Author relationship
[281] WO2020081010A1<br>Priority: 19 Oct 2018, SG10201809262T.	Applicant: National University of Singapore.	Textile wireless networking and externally supplied power; metamaterial pathways for radio-wave confinement and body-worn nodes.	Not every embodiment is battery-free. External RF supply is not autonomous energy harvesting.	No current manuscript author identified as inventor.
[282] WO2016150749A1<br>Priority: 23 Mar 2015, EP15160262.0.	Applicant: Koninklijke Philips N.V.	Skin-contact optical vital-sign sensing; source, detector, colour/wavelength conversion and interface packaging.	Does not independently establish heart-rate or peripheral oxygen-saturation accuracy, skin-tone robustness, clearance or clinical effectiveness.	No current manuscript author identified as inventor.
